# Graded Hoare Logic and its Categorical Semantics

**DOI:** 10.1007/978-3-030-72019-3_9

**Published:** 2021-03-23

**Authors:** Marco Gaboardi, Shin-ya Katsumata, Dominic Orchard, Tetsuya Sato

**Affiliations:** Imperial College, London, UK; 9 Boston University, Boston, USA; 10 National Institute of Informatics, Tokyo, Japan; 11 University of Kent, Canterbury, United Kingdom; 12 Tokyo Institute of Technology, Tokyo, Japan

## Abstract

Deductive verification techniques based on program logics (i.e., the family of Floyd-Hoare logics) are a powerful approach for program reasoning. Recently, there has been a trend of increasing the expressive power of such logics by augmenting their rules with additional information to reason about program side-effects. For example, general program logics have been augmented with cost analyses, logics for probabilistic computations have been augmented with estimate measures, and logics for differential privacy with indistinguishability bounds. In this work, we unify these various approaches via the paradigm of *grading*, adapted from the world of functional calculi and semantics. We propose *Graded Hoare Logic* (GHL), a parameterisable framework for augmenting program logics with a preordered monoidal analysis. We develop a semantic framework for modelling GHL such that grading, logical assertions (pre- and post-conditions) and the underlying effectful semantics of an imperative language can be integrated together. Central to our framework is the notion of a *graded category* which we extend here, introducing *graded Freyd categories* which provide a semantics that can interpret many examples of augmented program logics from the literature. We leverage coherent fibrations to model the base assertion language, and thus the overall setting is also fibrational.

### 1 Introduction

The paradigm of *grading* is an emerging approach for augmenting language semantics and type systems with fine-grained information [40]. For example, a *graded monad* provides a mechanism for embedding side-effects into a pure language, exactly as in the approach of monads, but where the types are augmented ("graded") with information about what effects may occur, akin to a type-and-effect system [24,42]. As another example, *graded comonadic* type operators in linear type systems can capture non-linear dataflow and properties of data use [7,16,44]. In general, graded types augment a type system with some algebraic structure which serves to give a parameterisable fine-grained program analysis capturing the underlying structure of a type theory or semantics.

Much of the work in graded types has arisen in conjunction with categorical semantics, in which graded modal type operators are modelled via graded monads [13,17,25,36,33], graded comonads (often with additional graded monoidal structure) [7,16,25,43,44], graded 'joinads' [36], graded distributive laws between graded (co)monads [15], and graded Lawvere theories [27].

So far grading has mainly been employed to reason about functional languages and calculi, thus the structure of the $\lambda$-calculus has dictated the structure of categorical models (although some recent work connects graded monads with classical dataflow analyses on CFGs [21]). We investigate here the paradigm of grading instead applied to *imperative* languages. As it happens, there is already a healthy thread of work in the literature augmenting program logics (in the family of Floyd-Hoare logics) with analyses that resemble notions of grading seen more recently in the functional world. The general approach is to extend the power of deductive verification by augmenting program logic rules with an analysis of side effects, tracked by composing rules. For example, work in the late 1980s and early 1990s augmented program logics with an analysis of computation time, accumulating a cost measure [37,38], with more recent fine-grained resource analysis based on multivariate analysis associated to program variables [8]. As another example, the Union Bound Logic of Barthe et al. [5] defines a Hoare-logic-style system for reasoning about probabilistic computations with judgments $\vdash_\beta c : \phi \Rightarrow \psi$ for a program $c$ annotated by the maximum probability $\beta$ (the union bound) that $\psi$ does not hold. The inference rules of Union Bound Logic track and compute the union bound alongside the standard rules of Floyd-Hoare logic. As a last example, Approximate Relational Hoare Logic [2,6,39,48] augments a program logic with measures of the $\epsilon$-$\delta$ bounds for reasoning about differential privacy.

In this work, we show how these disparate approaches can be unified by adapting the notion of grading to an imperative program-logic setting, for which we propose *Graded Hoare Logic* (GHL): a parameterisable program logic and reasoning framework graded by a preordered monoidal analysis. Our core contribution is GHL's underlying semantic framework which integrates grading, logical assertions (pre- and post-conditions) and the effectful semantics of an imperative language. This framework allows us to model, in a uniform way, the different augmented program logics discussed above.

Graded models of functional calculi tend to adopt either a graded monadic or graded comonadic model, depending on the direction of information flow in the analysis. We use the opportunity of an imperative setting (where the $\lambda$-calculus' asymmetrical 'many-inputs-to-one-output' model is avoided) to consider a more flexible semantic basis of *graded categories*. Graded categories generalise graded (co)monadic approaches, providing a notion of graded denotation without imposing on the placement (or 'polarity') of grading.

*Outline* Section 2 begins with an overview of the approach, focusing on the example of Union Bound Logic and highlighting the main components of our semantic framework. The next three sections then provide the central contributions:

– Section 3 defines GHL and its associated assertion logic which provides a flexible, parameterisable program logic for integrating different notions of side-effect reasoning, parameterised by a preordered monoidal analysis. We instantiate the program logic to various examples.
– Section 4 explores graded categories, an idea that has not been explored much in the literature, and for which there exists various related but not-quite-overlapping definitions. We show that graded categories can abstract graded monadic and graded comonadic semantics. We then extend graded categories to Freyd categories (generally used as a more flexible model of effects than monads), introducing the novel structure of *graded Freyd categories*.
– Section 5 develops the semantic framework for GHL, based on graded Freyd categories in a fibrational setting (where *coherent fibrations* [22] model the assertion logic) integrated with the graded Freyd layer. We instantiate the semantic model to capture the examples presented in Section 3 and others drawn from the literature mentioned above.

An extended version of this paper provides appendices which include further examples and proof details [14].

### 2 Overview of GHL and Prospectus of its Model

As discussed in the introduction, several works explore Hoare logics combined with some form of implicit or explicit grading for program analysis. Our aim is to study these in a uniform way. We informally introduce of our approach here.

We start with an example which can be derived in Union Bound Logic [5]:

$$\vdash_{0.05} \{\top\}\, \mathtt{do}\, v_1 \leftarrow \mathtt{Gauss}(0,1); \mathtt{do}\, v_2 \leftarrow \mathtt{Gauss}(0,1); v := \mathtt{max}(v_1, v_2)\, \{v \leq 2\}$$

This judgment has several important components. First, we have primitives for *procedures with side-effects* such as $\mathtt{do}\, v_1 \leftarrow \mathtt{Gauss}(0,1)$. This procedure samples a random value from the standard normal distribution with mean 0 and variance 1 and stores the result in the variable $v_1$. This kind of procedure with side effects differs from a regular assignment such as $v := \mathtt{max}(v_1, v_2)$, which is instead considered to be pure (wrt. probabilities) in our approach.

The judgment has grade '0.05' which expresses a bound on the probability that the postcondition is false, under the assumption of the precondition, after executing the program; we can think of it as the probability of failing to guarantee the postcondition. In our example (call it program $P$), since the precondition is true, this can be expressed as: $\Pr_{[\![P]\!](m)}[v > 2] \leq 0.05$ where $[\![P]\!](m)$ is the probability distribution generated in executing the program. The grade of $P$ in this logic is derived using three components. First, sequential composition:

$$\frac{\vdash_{\beta} \{\psi\}\, P_1\, \{\psi_1\} \qquad \vdash_{\beta'} \{\psi_1\}\, P_2\, \{\phi\}}{\vdash_{\beta+\beta'} \{\psi\}\, P_1; P_2\, \{\phi\}}$$

which sums the failure probabilities. Second, an axiom for Gaussian distribution:

$$\vdash_{0.025} \{\top\}\, \mathtt{do}\, v \leftarrow \mathtt{Gauss}(0,1)\, \{v \leq 2\}$$

with a basic constant 0.025 which comes from the property of the Gaussian distribution we are considering. Third, by the following judgment which is derivable by the assignment and the consequence rules, which are the ones from Hoare Logic with a trivial grading 0 which is the unit of addition:

$$\vdash_0 \{v_1 \leq 2 \vee v_2 \leq 2\}\ v := \mathtt{max}(v_1, v_2)\ \{v \leq 2\}$$

Judgments for more complex examples can be derived using the rules for conditional and loops. These rules also consider grading, and the grading can depend on properties of the program. For example the rule for conditionals is:

$$\frac{\vdash_\beta \{\psi \wedge e_b = \mathtt{tt}\}\ P_1\ \{\phi\} \qquad \vdash_\beta \{\psi \wedge e_b = \mathtt{ff}\}\ P_2\ \{\phi\}}{\vdash_\beta \{\psi\}\ \mathtt{if}\ e_b\ \mathtt{then}\ P_1\ \mathtt{else}\ P_2\ \{\phi\}}$$

This allows one to reason also about the grading in a conditional way, through the two assumptions $\psi \wedge e_b = \mathtt{tt}$ and $\psi \wedge e_b = \mathtt{ff}$. We give more examples later.

Other logics share a similar structure as that described above for the Union Bound logic, for example the relational logic apRHL [2], and its variants [48,49], for reasoning about differential privacy. Others again use a similar structure implicitly, for example the Hoare Logic to reason about asymptotic execution cost by Nielson [37], Quantitative Hoare Logic [8], or the relational logic for reasoning about program counter security presented by Barthe [3].

To study the semantics of these logics in a uniform way, we first abstract the logic itself. We design a program logic, which we call Graded Hoare Logic (GHL), containing all the components discussed above. In particular, the language is a standard imperative language with conditional and loops. Since our main focus is studying the semantics of grading, for simplicity we avoid using a 'while' loop, using instead a bounded 'loop' operation ($\mathtt{loop}\, e\ \mathtt{do}\ P$). This allow us to focus on the grading structures for total functions, leaving the study of the interaction between grading and partiality to future work. The language is parametric in the operations that are supported in expressions—common in several treatments of Hoare Logic—and in a set of procedures and commands with side effects, which are the main focus of our work. GHL is built over this language and an *assertion logic* which is parametric in the basic predicates that can be used to reason about programs. GHL is also parametric in a preordered monoid of grades, and in the axioms associated with basic procedures and commands with side effects. This generality is needed in order to capture the different logics we mentioned before.

GHL gives us a unified syntax, but our real focus is the semantics. To be as general as possible we turn to the language of category theory. We give a categorical framework which can capture different computational models and side effects, with denotations that are refined by predicates and grades describing program behaviours. Our framework relates different categories (modelling different aspects of GHL) as summarized by the following informal diagram (1).

$$\begin{array}{ccc} \mathbb{P} & \xrightarrow{\dot{I}} & \mathbb{E} \\ {\scriptstyle p}\downarrow & & \downarrow{\scriptstyle q} \\ \mathbb{V} & \xrightarrow{I} & \mathbb{C} \end{array} \qquad (1)$$

This diagram should not be understood as a commutative diagram in **CAT** as $\mathbb{E}$ is a graded category and hence not an object of **CAT**.

The category $\mathbb{V}$ models values and pure computations, the category $\mathbb{C}$ models impure computations, $\mathbb{P}$ is a category of predicates, and $\mathbb{E}$ is a *graded category* whose hom-sets are indexed by *grades*—elements of a preordered monoid. The presentation of graded categories is new here, but has some relation to other structures of the same name (discussed in Section 4).

This diagram echos the principle of *refinement as functors* proposed by Melliès and Zeilberger [32]. The lower part of the diagram offers an interpretation of the language, while the upper part offers a logical refinement of programs with grading. However, our focus is to introduce a new *graded refinement* view. The ideas we use to achieve this are to interpret the base imperative language using a *Freyd category* $I : \mathbb{V} \to \mathbb{C}$ (traditionally used to model effects) with countable coproducts, to interpret the assertion logic with a *coherent fibration* $p : \mathbb{P} \to \mathbb{V}$, and to interpret GHL as a *graded Freyd category* $\dot{I} : \mathbb{P} \to \mathbb{E}$ with homogeneous coproducts. In addition, the graded category $\mathbb{E}$ has a functor[5] $q$ into $\mathbb{C}$ which erases assertions and grades and extracts the denotation of effectful programs, in the spirit of refinements. The benefit of using a Freyd category as a building block is that they are more flexible than other structures (e.g., monads) for constructing models of computational effects [47,51]. For instance, in the category **Meas** of measurable spaces and measurable functions, we cannot define state monads since there are no exponential objects. However, we can still have a model of first-order effectful computations using Freyd categories [46].

Graded Freyd categories are a new categorical structure that we designed for interpreting GHL judgments (Section 4.2). The major difference from an ordinary Freyd category is that the 'target' category is now a *graded category* ($\mathbb{E}$ in the diagram (1)). The additional structure provides what we need in order to interpret judgments including grading.

To show the generality of this structure, we present several approaches to instantiating the categorical framework of GHL's semantics, showing constructions via graded monads and graded comonads preserving coproducts.

Part of the challenge in designing a categorical semantics for GHL is to carve out and implement the implicit assumptions and structures used in the semantics of the various Hoare logics. A representative example of this challenge is the interpretation of the rule for conditionals in Union Bound Logic that we introduced above. We interpret the assertion logic in (a variant of) coherent fibrations $p : \mathbb{P} \to \mathbb{V}$, which model the $\wedge\vee\exists{=}$-fragment of first-order predicate logic [22]. In this abstract setup, the rule for conditionals may become *unsound* as it is built on the implicit assumption that the type $\mathsf{Bool}$, which is interpreted as $1+1$, consists only of two elements, but this may fail in general $\mathbb{V}$. For example, a suitable coherent fibration for relational Hoare logic would take $\mathbf{Set}^2$ as the base category, but we have $\mathbf{Set}^2(1, 1+1) \cong 4$, meaning that there are four global elements in the interpretation of $\mathsf{Bool}$. We resolve this problem by introducing

[5] More precisely, this is not quite a functor because $\mathbb{E}$ is a graded category; see Definition 9 for the precise meaning.

a side condition to guarantee the decidability of the boolean expression:

$$\frac{\vdash_m \{\psi \wedge e_b = \mathtt{tt}\}\, P_1\, \{\phi\} \quad \vdash_m \{\psi \wedge e_b = \mathtt{ff}\}\, P_2\, \{\phi\} \quad \psi \vdash e_b = \mathtt{tt} \vee e_b = \mathtt{ff}}{\vdash_m \{\psi\}\, \mathtt{if}\, e_b\, \mathtt{then}\, P_1\, \mathtt{else}\, P_2\, \{\phi\}}$$

This is related to the synchronization condition appearing in the relational Hoare logic rule for conditional commands (e.g., [6]).

Another challenge in the design of the GHL is how to assign a grade to the loop command $\mathtt{loop}\, e\, \mathtt{do}\, P$. We may naïvely give it the grade $m_l \triangleq \bigvee_{i \in \mathbb{N}} m^i$, where $m$ is the grade of $P$, because $P$ is repeatedly executed some finite number of times. However, the grade $m_l$ is a very loose over-approximation of the grade of $\mathtt{loop}\, e\, \mathtt{do}\, P$. Even if we obtain some knowledge about the iteration count $e$ in the assertion logic, this cannot be reflected in the grade. To overcome this problem, we introduce a Hoare logic rule that can estimate a more precise grade of $\mathtt{loop}\, e\, \mathtt{do}\, P$, provided that the value of $e$ is determined:

$$\frac{\forall 0 \leq z < N.\ \vdash_m \{\psi_{z+1}\}\, P\, \{\psi_z\} \quad \psi_N \vdash e_n = \lceil N \rceil}{\vdash_{m^N} \{\psi_N\}\, \mathtt{loop}\, e_n\, \mathtt{do}\, P\, \{\psi_0\}}$$

This rule brings together the assertion language and grading, creating a dependency from the former to the latter, and giving us the structure needed for a categorical model. The right premise is a judgment of the assertion logic (under program variables $\Gamma_{\mathsf{M}}$ and pre-condition $\psi_N$) requiring that $e$ is statically determinable as $N$. This premise makes the rule difficult to use in practical applications where $e$ is dynamic. We expect a more "dependent" version of this rule is possible with a more complex semantics internalizing some form of data-dependency. Nevertheless, the above is enough to study the semantics of grading and its interaction with the Hoare Logic structure, which is our main goal here.

### 3 Loop Language and Graded Hoare Logic

After introducing some notation and basic concepts used throughout, we outline a core imperative loop language, parametric in its set of basic commands and procedures (Section 3.2). We then define a template of an assertion logic (Section 3.3), which is the basis of Graded Hoare Logic (Section 3.4).

#### 3.1 Preliminaries

Throughout, we fix an infinite set **Var** of variables which are employed in the loop language (as the names of mutable program variables) and in logic (to reason about these program variables).

A *many-sorted signature* $\Sigma$ is a tuple $(S, O, ar)$ where $S, O$ are sets of sorts and operators, and $ar : O \to S^+$ assigns argument sorts and a return value sort to operators (where $S^+$ is a non-empty sequence of sorts, i.e., an operator $o$ with signature $(s_1 \times \ldots \times s_n) \to s$ is summarized as $ar(o) = \langle s_1, \ldots, s_n, s \rangle \in S^+$). We say that another many-sorted signature $\Sigma' = (S', O', ar')$ is an *extension* of $\Sigma$ if $S \subseteq S'$ and $O \subseteq O'$ and $ar(o) = ar'(o)$ for all $o \in O$.

Let $\Sigma = (S, \cdots)$ be a many-sorted signature. A *context* for $\Sigma$ is a (possibly empty) sequence of pairs $\Gamma \in (\mathbf{Var} \times S)^*$ such that all variables in $\Gamma$ are distinct. We regard $\Gamma$ as a partial mapping from $\mathbf{Var}$ to $S$. The set of contexts for $\Sigma$ is denoted $\mathbf{Ctx}_\Sigma$. For $s \in S$ and $\Gamma \in \mathbf{Ctx}_\Sigma$, we denote by $\mathbf{Exp}_\Sigma(\Gamma, s)$ the set of $\Sigma$-expressions of sort $s$ under the context $\Gamma$. When $\Sigma, \Gamma$ are obvious, we simply write $e : s$ to mean $e \in \mathbf{Exp}_\Sigma(\Gamma, s)$. This set is inductively defined as usual.

An *interpretation* of a many-sorted signature $\Sigma = (S, O, ar)$ in a cartesian category $(\mathbb{V}, 1, \times)$ consists of an assignment of an object $[\![s]\!] \in \mathbb{V}$ for each sort $s \in S$ and an assignment of a morphism $[\![o]\!] \in \mathbb{V}([\![s_1]\!] \times \cdots \times [\![s_n]\!], [\![s]\!])$ for each $o \in O$ such that $ar(o) = \langle s_1, \ldots, s_n, s \rangle$. Once such an interpretation is given, we extend it to $\Sigma$-expressions in the standard way (see, e.g. [9,45]). First, for a context $\Gamma = x_1 : s_1, \cdots, x_n : s_n \in \mathbf{Ctx}_\Sigma$, by $[\![\Gamma]\!]$ we mean the product $[\![s_1]\!] \times \cdots \times [\![s_n]\!]$. Then we inductively define the interpretation of $e \in \mathbf{Exp}_\Sigma(\Gamma, s)$ as a morphism $[\![e]\!] \in \mathbb{V}([\![\Gamma]\!], [\![s]\!])$.

Throughout, we write bullet-pointed lists marked with $\star$ for the mathematical data that are parameters to Graded Hoare Logic (introduced in Section 3.4).

#### 3.2 The Loop Language

We introduce an imperative language called the loop language, with a finite looping construct. The language is parameterised by the following data:

- $\star$ a many-sorted signature $\Sigma = (S, O, ar)$ extending a base signature $(S_0, O_0, ar_0)$ of sort $S_0 = \{\texttt{bool}, \texttt{nat}\}$ with essential constants as base operators $O_0$, shown here with their signatures for brevity rather than defining $ar_0$ directly:

$$O_0 = \{\texttt{tt} : \texttt{bool}, \texttt{ff} : \texttt{bool}\} \cup \{\lceil k \rceil : \texttt{nat} \mid k \in \mathbb{N}\}$$

where `bool` is used for branching control-flow and `nat` is used for controlling loops, whose syntactic constructs are given below. We write $\lceil k \rceil$ to mean the embedding of semantic natural numbers into the syntax.
- $\star$ a set $\mathbf{CExp}$ of *command names* (ranged over by $c$) and a set $\mathbf{PExp}_s$ of *procedure names of sort* $s$ (ranged over by $p$) for each sort $s \in S$.

When giving a program, we first fix a context $\Gamma_\mathsf{M}$ for the program variables. We define the set of *programs* (under a context $\Gamma_\mathsf{M}$) by the following grammar:

$$P ::= P\,;P \mid \texttt{skip} \mid v := e \mid \texttt{do}\,c \mid \texttt{do}\,v \leftarrow p \mid \texttt{if}\,e_b\,\texttt{then}\,P\,\texttt{else}\,P \mid \texttt{loop}\,e_n\,\texttt{do}\,P$$

where $v \in \Gamma_\mathsf{M}$, $e_b, e_n$ are well-typed $\Sigma$-expressions of sort `bool` and `nat` under $\Gamma_\mathsf{M}$, and $c \in \mathbf{CExp}$. In assignment commands, $e \in \mathbf{Exp}_\Sigma(\Gamma_\mathsf{M}, \Gamma(v))$. In procedure call commands, $p \in \mathbf{PExp}_{\Gamma(v)}$. Each program must be well-typed under $\Gamma_\mathsf{M}$. The typing rules are routine so we omit them.

Thus, programs can be sequentially composed via ; with `skip` as the trivial program which acts as a unit to sequencing. An assignment $v := e$ assigns expressions to a program variable $v$. Commands can be executed through the instruction $\texttt{do}\,c$ which yields some side effects but does not return any value.

Procedures can be executed through a similar instruction $\texttt{do}\, v \leftarrow p$ which yields some side effect but also returns a value which is used to update $v$. Finally, conditionals are guarded by a boolean expression $e_b$ and the iterations of a looping construct are given by a natural number expression $e_n$ (which is evaluated once at the beginning of the loop to determine the number of iterations).

This language is rather standard, except for the treatment of commands and procedures of which we give some examples here.

*Example 1. Cost Information:* a simple example of a command is `tick`, which yields as a side effect the recording of one 'step' of computation.

*Control-Flow Information:* two other simple example of commands are `cfTT` and `cfFF`, which yield as side effects the recording of either true or false to a log. A program can be augmented with these commands in its branches to give an account of a program's control flow. We will use these commands to reason about control-flow security in Example 3.

*Probability Distributions:* a simple example of a procedure is $\texttt{Gauss}(x, y)$, which yields as a side effect the introduction of new randomness in the program, and which returns a random sample from the Gaussian distribution with mean and variance specified by $x, y \in \Gamma_{\mathsf{M}}$. We will see how to use this procedure to reason about probability of failure in Example 4.

Concrete instances of the loop language typically include conversion functions between the sorts in $\Sigma$, e.g., so that programs can dynamically change control flow depending on values of program variables. In other instances, we may have a language manipulating richer data types, e.g., reals or lists, and also procedures capturing higher-complexity computations, such as Ackermann functions.

#### 3.3 Assertion Logic

We use an assertion logic to reason about properties of basic expressions. We regard this reasoning as a meta-level activity, thus the logic can have more sorts and operators than the loop language. Thus, over the data specifying the loop language, we build formulas of the assertion logic by the following data:

- ⋆ a many-sorted signature $\Sigma_l = (S_l, O_l, ar_l)$ extending $\Sigma$.
- ⋆ a set $P_l$ of atomic propositions and a function $par_l : P_l \to S_l^*$ assigning input sorts to them. We then inductively define the set $\mathbf{Fml}_{\Sigma_l}(\Gamma)$ of formulas under $\Gamma \in \mathbf{Ctx}_{\Sigma_l}$ as in Figure 1 (over the page), ranged over by $\psi$ and $\phi$.
- ⋆ a $\mathbf{Ctx}_{\Sigma_l}$-indexed family of subsets $\mathbf{Axiom}(\Gamma) \subseteq \mathbf{Fml}_{\Sigma_l}(\Gamma) \times \mathbf{Fml}_{\Sigma_l}(\Gamma)$.

The assertion logic is a fragment of the many-sorted first-order logic over $\Sigma_l$-terms admitting: 1) finite conjunctions, 2) countable disjunctions, 3) existential quantification, and 4) equality predicates. Judgements in the assertion logic have the form $\Gamma \mid \psi_1, \cdots, \psi_n \vdash \phi$ (read as $\psi_1 \wedge \cdots \wedge \psi_n$ implies $\phi$), where $\Gamma \in \mathbf{Ctx}_{\Sigma_l}$ is a context giving types to variables in the formulas $\psi_1, \cdots, \psi_n, \phi \in \mathbf{Fml}_{\Sigma_l}(\Gamma)$. The logic has the axiom rule deriving $\Gamma \mid \psi \vdash \phi$ for each pair $(\psi, \phi)$ of formulas in $\mathbf{Axiom}(\Gamma)$. The rest of inference rules of this logic are fairly standard and so we omit them (see e.g. [22, Section 3.2 and Section 4.1]).

The set $\mathbf{Fml}_{\Sigma_l}(\Gamma)$ of formulas under $\Gamma \in \mathbf{Ctx}_{\Sigma_l}$ is inductively defined as follows:

1. For all $p \in P_l$ and $par_l(p) = s_1 \cdots s_n$ and $t_i : \mathbf{Exp}_{\Sigma_l}(\Gamma, s_i)$ $(1 \le i \le n)$ implies $p(t_1, \cdots, t_n) \in \mathbf{Fml}_{\Sigma_l}(\Gamma)$
2. For all $s \in S_l$ and $t, u \in \mathbf{Exp}_{\Sigma_l}(\Gamma, s)$, $t = u \in \mathbf{Fml}_{\Sigma_l}(\Gamma)$.
3. For all finite families $\{\phi_i \in \mathbf{Fml}_{\Sigma_l}(\Gamma)\}_{i \in \Lambda}$, we have $\bigwedge \phi_i \in \mathbf{Fml}_{\Sigma_l}(\Gamma)$.
4. For all countable families $\{\phi_i \in \mathbf{Fml}_{\Sigma_l}(\Gamma)\}_{i \in \Lambda}$, we have $\bigvee \phi_i \in \mathbf{Fml}_{\Sigma_l}(\Gamma)$.
5. For all $\phi \in \mathbf{Fml}_{\Sigma_l}(\Gamma, x : s)$, we have $(\exists x : s \,.\, \phi) \in \mathbf{Fml}_{\Sigma_l}(\Gamma)$.

**Fig. 1.** Formula formation rules

In some of our examples we will use the assertion logic to reason about programs in a relational way, i.e., to reason about two executions of a program (we call them *left* and *right* executions). This requires basic predicates to manage expressions representing pairs of values in our assertion logic. As an example, we could have two predicates $\mathsf{eqv}_{\langle 1 \rangle}$, $\mathsf{eqv}_{\langle 2 \rangle}$, that can assert the equality of the left and right executions of an expression to some value, respectively. That is, the formula $\mathsf{eqv}_{\langle 1 \rangle}(e_b, \mathsf{true})$, which we will write using infix notation $e_b\langle 1 \rangle = \mathsf{true}$, asserts that the left execution of the boolean expression $e_b$ is equal to $\mathsf{true}$.

#### 3.4 Graded Hoare Logic

We now introduce Graded Hoare Logic (GHL), specified by the following data:

- ⋆ a preordered monoid $(M, \le, 1, \cdot)$ (*pomonoid* for short) (where $\cdot$ is monotonic with respect to $\le$) for the purposes of program analysis, where we refer to the elements $m \in M$ as *grades*;
- ⋆ two functions which define the grades and pre- and post-conditions of commands **CExp** and procedures **PExp**:

$$C_{\mathsf{c}} : \mathbf{Fml}_{\Sigma_l}(\Gamma_{\mathsf{M}}) \times M \to 2^{\mathbf{CExp}}$$
$$C^s_{\mathsf{p}} : \mathbf{Fml}_{\Sigma_l}(\Gamma_{\mathsf{M}}) \times M \times \mathbf{Fml}_{\Sigma_l}(r : s) \to 2^{\mathbf{PExp}_s} \quad (s \in S \wedge r \notin \mathsf{dom}(\Gamma_{\mathsf{M}}))$$

The function $C_{\mathsf{c}}$ takes a pre-condition and a grade, returning a set of command symbols satisfying these specifications. A command $c$ may appear in $C_{\mathsf{c}}(\phi, m)$ for different pairs $(\phi, m)$, enabling pre-condition-dependent grades to be assigned to $c$. Similarly, the function $C^s_{\mathsf{p}}$ takes a pre-condition, a grade, and a postcondition for return values, and returns a set of procedure names of sort $s$ satisfying these specifications. Note, $r$ is a distinguished variable (for return values) not in $\Gamma_{\mathsf{M}}$. The shape of $C_{\mathsf{c}}$ and $C_{\mathsf{c}}$ as predicates over commands and procedures, indexed by assertions and grades, provides a way to link grades and assertions for the effectful operations of GHL. Section 3.5 gives examples exploiting this.

From this structure we define a *graded Hoare logic* by judgments of the form: $\vdash_m \{\phi\}\ P\ \{\psi\}$ denoting a program $P$ with pre-condition $\phi \in \mathbf{Fml}_{\Sigma_l}(\Gamma_{\mathsf{M}})$, post-condition $\psi \in \mathbf{Fml}_{\Sigma_l}(\Gamma_{\mathsf{M}})$ and analysis $m \in M$. Graded judgments are defined inductively via the inference rules given in Table 1. Ignoring grading, many of the rules are fairly standard for a Floyd-Hoare program logic. The rule for `skip` is standard but includes grading by the unit 1 of the monoid. Similarly, assignment

$$\frac{}{\vdash_1 \{\psi\}\, \texttt{skip}\, \{\psi\}} \qquad \frac{\vdash_m \{\psi\}\, P_1\, \{\psi_1\} \quad \vdash_{m'} \{\psi_1\}\, P_2\, \{\phi\}}{\vdash_{m \cdot m'} \{\psi\}\, P_1; P_2\, \{\phi\}} \qquad \frac{}{\vdash_1 \{\psi[e/v]\}\, v := e\, \{\psi\}}$$

$$\frac{f \in C_{\mathsf{c}}(\psi, m)}{\vdash_m \{\psi\}\, \texttt{do}\, c\, \{\psi\}} \qquad \frac{p \in C_{\mathsf{p}}^{\Gamma_{\mathsf{M}}(v)}(\psi, m, \phi)}{\vdash_m \{\psi\}\, \texttt{do}\, v \leftarrow p\, \{(\exists v : \Gamma_{\mathsf{M}}(v)\ .\ \psi) \wedge \phi[v/r]\}}$$

$$\frac{\Gamma_{\mathsf{M}} \mid \psi' \vdash \psi \quad m \leq m' \quad \Gamma_{\mathsf{M}} \mid \phi \vdash \phi' \quad \vdash_m \{\psi\}\, P\, \{\phi\}}{\vdash_{m'} \{\psi'\}\, P\, \{\phi'\}}$$

$$\frac{\forall 0 \leq z < N.\ \vdash_m \{\psi_{z+1}\}\, P\, \{\psi_z\} \quad \Gamma_{\mathsf{M}} \mid \psi_N \vdash e_n = \lceil N \rceil}{\vdash_{m^N} \{\psi_N\}\, \texttt{loop}\, e_n\, \texttt{do}\, P\, \{\psi_0\}}$$

$$\frac{\vdash_m \{\psi \wedge e_b = \texttt{tt}\}\, P_1\, \{\phi\} \quad \vdash_m \{\psi \wedge e_b = \texttt{ff}\}\, P_2\, \{\phi\} \quad \Gamma_{\mathsf{M}} \mid \psi \vdash e_b = \texttt{tt} \vee e_b = \texttt{ff}}{\vdash_m \{\psi\}\, \texttt{if}\, e_b\, \texttt{then}\, P_1\, \texttt{else}\, P_2\, \{\phi\}}$$

**Table 1.** Graded Hoare Logic Inference Rules is standard, but graded with 1 since we do not treat it specially in GHL. Sequential composition takes the monoid multiplication of the grades of the subterms. The rules for commands and procedures use the functions $C_{\mathsf{c}}$ and $C_{\mathsf{p}}$ introduced above. Notice that the rule for commands uses as the pre-condition as its post-condition, since commands have only side effects and they do not return any value. The rule for procedures combines the pre- and post-conditions given by $C_{\mathsf{p}}$ following the style of Floyd's assignment rule [12].

The non-syntax-directed consequence rule is similar to the usual consequence rule, and in addition allows the assumption on the grade to be weakened (*approximated*) according to the ordering of the monoid.

The shape of the loop rule is slightly different from the usual one. It uses the assertion-logic judgment $\Gamma_{\mathsf{M}} \mid \psi_N \vdash e_n = \lceil N \rceil$ to express the assumption that $e_n$ evaluates to $\lceil N \rceil$. Under this assumption it uses a family of assertions $\psi_z$ indexed by the natural numbers $z \in \{0, 1, \ldots, N-1\}$ to conclude the post-condition $\psi_0$. This family of assertions plays the role of the classical invariant in the Floyd-Hoare logic rule for 'while'. Assuming that the grade of the loop body is $m$, the grade of the loop command is then $m^N$, where $m^0 = 1$ and $m^{k+1} = m \cdot m^k$. By instantiating this rule with $\psi_z = (\theta \wedge e_n = \lceil z \rceil)$, the loop rule also supports the following derived rule which is often preferable in examples:

$$\frac{\forall 0 \leq z < N.\ \vdash_m \{\theta \wedge e_n = \lceil z+1 \rceil\}\, P\, \{\theta \wedge e_n = \lceil z \rceil\}}{\vdash_{m^N} \{\theta \wedge e_n = \lceil N \rceil\}\, \texttt{loop}\, e_n\, \texttt{do}\, P\, \{\theta \wedge e_n = \lceil 0 \rceil\}}$$

The rule for the conditional is standard except for the condition $\Gamma_{\mathsf{M}} \mid \psi \vdash e_b = \texttt{tt} \vee e_b = \texttt{ff}$. While this condition may seem obvious, it is actually important to make GHL sound in various semantics (mentioned in Section 2). As an example, suppose that a semantics $[\![-]\!]$ of expressions is given in the product category $\mathbf{Set}^2$, which corresponds to two semantics $[\![-]\!]_1, [\![-]\!]_2$ of expressions in $\mathbf{Set}$. Then the side condition for the conditional is to guarantee that for any boolean expression $e_b$, and pair of memories $(\rho_1, \rho_2)$ satisfying the precondition $\psi$, the pair $(\llbracket e_b \rrbracket_1(\rho_1), \llbracket e_b \rrbracket_2(\rho_2))$ is either $\llbracket \texttt{tt} \rrbracket = (\mathsf{tt}, \mathsf{tt})$ or $\llbracket \texttt{ff} \rrbracket = (\mathsf{ff}, \mathsf{ff})$. We note that other relational logics such as apRHL [6] employ an equivalent syntactic side condition in their rule for conditionals.

#### 3.5 Example Instantiations of GHL

*Example 2 (Simple cost analysis).* We can use the `tick` command discussed in Example 1 to instrument programs with *cost* annotations. We can then use GHL to perform cost analysis by instantiating GHL with the additive natural number monoid $(\mathbb{N}, \leq, 0, +)$ and $\texttt{tick} \in C_{\mathsf{c}}(\phi, 1)$. Thus, we can form judgments $\vdash_1 \{\phi\}\, \texttt{do}\, \texttt{tick}\, \{\phi\}$ which account for cost via the judgment's grade. Sequential composition accumulates cost and terms like `skip` and assignment have 0 cost.

Let us use this example to illustrate how $C_{\mathsf{c}}$ can assign multiple pre-condition-grade pairs to a command. Suppose that we modify the semantics of `tick` so that it reports unit cost 1 when variable $x$ is 0, otherwise cost 2. We can then define $C_{\mathsf{c}}$ so that $\texttt{tick} \in C_{\mathsf{c}}(x = \lceil 0 \rceil, 1)$ and also $\texttt{tick} \in C_{\mathsf{c}}(x \neq \lceil 0 \rceil, 2)$. In this way, we can give different grades to programs depending on their pre-conditions.

*Example 3 (Program Counter Security).* We can use the commands `cfTT` and `cfFF` discussed in Example 1 to instrument programs with *control flow* annotations, recording to an external log. GHL can then be used to reason about program counter security [35][3, Section 7.2] of instrumented programs. This is a relational security property similar to non-interference (requiring that private values do not influence public outputs) but where only programs with the same control flow are considered.

Firstly, any conditional statement $\texttt{if}\, e_b\, \texttt{then}\, P_t\, \texttt{else}\, P_f$ in a program is elaborated to a statement $\texttt{if}\, e_b\, \texttt{then}\, (\texttt{cfTT}; P_t)\, \texttt{else}\, (\texttt{cfFF}; P_f)$. We then instantiate GHL with a monoid of words over $\{\texttt{tt}, \texttt{ff}\}$ with prefix order: $2^* \triangleq (\{\texttt{tt}, \texttt{ff}\}^*, \leq, \epsilon, \cdot)$ and we consider $\texttt{cfTT} \in C_{\mathsf{c}}(\phi, \texttt{tt})$ and $\texttt{cfTT} \in C_{\mathsf{c}}(\phi, \texttt{ff})$. We can thus form judgments of the shape $\vdash_{\texttt{tt}} \{\phi\}\, \texttt{do}\, \texttt{cfTT}\, \{\phi\}$ and $\vdash_{\texttt{ff}} \{\phi\}\, \texttt{do}\, \texttt{cfFF}\, \{\phi\}$ which account for control-flow information (forming paths) via the judgment's grade. Sequential composition concatenates control-flow paths and terms like `skip` and assignment do not provide any control-flow information, i.e. $\epsilon$.

We then instantiate the assertion logic to support relational reasoning, i.e., where the expressions of the language are interpreted as pair of values. For an expression $e$, interpreted as a pair $(v_1, v_2)$ then we write $e\langle 1 \rangle = v_1$ to say that the first component (left execution) equals $v_1$ and $e\langle 2 \rangle = v_2$ to say that the second component (right execution) equals $v_2$. In the assertion logic, we can then describe public values which need to be equal, following the tradition in reasoning about non-interference, by the predicate $e\langle 1 \rangle = e\langle 2 \rangle$. Private data are instead interpreted as a pair of arbitrary values. (Section 3.3 suggested the notation $\mathsf{eqv}_{\langle i \rangle}(e, b)$ for $e\langle i \rangle = b$, but we use the latter for compactness here).

As an example, one can prove the following judgment where $x$ is a public variable and $y$ is a private one, and $b \in \{\texttt{tt}, \texttt{ff}\}$:

$$\vdash_b \{x\langle 1\rangle{=}x\langle 2\rangle \wedge x\langle 1\rangle{=}b\}\,\texttt{if}\;x\;\texttt{then}\;(\texttt{cfTT};x{=}1;y{=}1)\;\texttt{else}\;(\texttt{cfFF};x{=}2;y{=}2)\{x\langle 1\rangle{=}x\langle 2\rangle\}$$

This judgment shows the program is non-interferent, since the value of $x$ is independent from the value of the private variable $y$, and secure in the program counter model, since the control flow does not depend on the value of $y$. Conversely, the following judgment is not derivable for both $b = \texttt{tt}$ and $b = \texttt{ff}$:

$$\vdash_b \{x\langle 1\rangle{=}x\langle 2\rangle \wedge y\langle 1\rangle{=}b\}\,\texttt{if}\;y\;\texttt{then}\;(\texttt{cfTT};x{=}1;y{=}1)\;\texttt{else}\;(\texttt{cfFF};x{=}1;y{=}2)\{x\langle 1\rangle{=}x\langle 2\rangle\}$$

This program is non-interferent but is not secure in the program counter model because the control flow leaks information about $y$ which is a private variable.

*Example 4 (Union Bound Logic).* Section 1 discussed the Union Bound logic by Barthe et al. [5]. This logic embeds smoothly into GHL by using the pomonoid $(\mathbb{R}_{\geq 0}, \leq, 0, +)$ and procedures of the form $\texttt{sample}_{\mu,e}$ as samplings from a probabilistic distribution $\mu$ parametrised over the syntax of GHL expressions $e$. Following Barthe et al. [5], we consider a semantically defined set for $C_p$:

$$C_p(\phi, \beta, \psi) = \{\texttt{sample}_{\mu,e} \mid \forall s.s \in [\![\phi]\!] \implies \Pr_{s' \leftarrow [\![\texttt{sample}_{\mu,e}]\!](s)}[s' \in [\![\neg\psi]\!] \leq \beta)\}$$

This definition captures that, assuming the pre-condition holds for an input memory state $s$, then for output value $s'$ from sampling $\texttt{sample}_{\mu,e}$, the probability that the post-condition is false is bounded above by $\beta$. This allow us to consider different properties of the distribution $\mu$ with parameter $e$.

### 4 Graded Categories

Now that we have introduced GHL and key examples, we turn to the core of its categorical semantics: *graded categories.*

Graded monads provide a notion of sequential composition for morphisms of the form $I \to T_m J$, i.e., with structure on the target/output capturing some information by the grade $m$ drawn from a pomonoid [24]; dually, graded comonads provide composition for $D_m I \to J$, i.e. with structure on the source/input with grade $m$ [43]. We avoid the choice of whether to associate grading with the input or output by instead introducing *graded categories*, which are agnostic about the polarity (or position) of any structure and grading. Throughout this section, we fix a pomonoid $(M, \leq, 1, \cdot)$ (with $\cdot$ monotonic wrt. $\leq$).

**Definition 1.** An *M-graded category* $\mathbb{C}$ consists of the following data:

- A class $\mathbf{Obj}(\mathbb{C})$ of objects. $I \in \mathbb{C}$ denotes $I \in \mathbf{Obj}(\mathbb{C})$.
- A homset $\mathbb{C}(I, J)(m)$ for all objects $I, J \in \mathbb{C}$ and $m \in M$. We often write $f : I \to_m J$ to mean $f \in \mathbb{C}(I, J)(m)$, and call $m$ the *grade* of $f$;
- An upcast functions $\uparrow_m^n : \mathbb{C}(I, J)(m) \to \mathbb{C}(I, J)(n)$ for all grades $m \leq n$;
- Identity morphisms $\mathrm{id}_I \in \mathbb{C}(I, I)(1)$ for all $I \in \mathbb{C}$;
- Composition $\circ : \mathbb{C}(J, K)(n) \times \mathbb{C}(I, J)(m) \to \mathbb{C}(I, K)(m \cdot n)$.

Graded categories satisfy the usual categorical laws of identity and associativity, and also the commutativity of upcast and composition: $\uparrow_n^{n'} g \circ \uparrow_m^{m'} f = \uparrow_{m \cdot n}^{m' \cdot n'} (g \circ f)$, corresponding to monotonicity of $(\cdot)$ with respect to $\leq$.

An intuitive meaning of a graded category's morphisms is: $f \in \mathbb{C}(A, B)(m)$ if the *value* or the *price* of a morphism $f : A \to B$ is *at most* $m$ with respect to the ordering $\leq$ on $M$. We do not yet give a polarity or direction to this price, i.e., whether the price is *consumed* or *produced* by the computation. Thus, graded categories give a non-biased view; we need not specify whether grading relates to the source or target of a morphism.

Graded categories were first introduced by Wood [54, Section 1] (under the name 'large $V$-categories'), and Levy connected them with models of call-by-push-value [28]. Therefore we do not claim the novelty of Definition 1.

*Example 5.* A major source of graded categories is via graded (co)monads. Let $(M, \leq, 1, \cdot)$ be a pomonoid, regarded as a monoidal category. A *graded monad* [50,24] on a category $\mathbb{C}$ (or more precisely an $M$*-graded monad*) is a lax monoidal functor $(T, \eta, \mu) : (M, \leq, 1, \cdot) \to ([\mathbb{C}, \mathbb{C}], \mathrm{Id}, \circ)$. Concretely, this specifies:

- a functor $T : (M, \leq) \to [\mathbb{C}, \mathbb{C}]$ from the preordered set $(M, \leq)$ to the endofunctor category over $\mathbb{C}$. For an ordered pair $m \leq m'$ in $M$ then $T(m \leq m') : Tm \to Tm'$ is a natural transformation;
- a unit $\eta : \mathrm{Id} \to T1$ and a multiplication $\mu_{m,m'} : Tm \circ Tm' \to T(m \cdot m')$, natural in $m, m' \in M$.

They satisfy the graded versions of the usual monad axioms:

$$\begin{array}{ccc} TmJ & \xrightarrow{Tm\eta_J} & Tm(T1J) \\ {\scriptstyle \eta_{TmJ}}\downarrow & = & \downarrow{\scriptstyle \mu_{m,1,J}} \\ T1(TmJ) & \xrightarrow{\mu_{1,m,J}} & TmJ \end{array} \qquad \begin{array}{ccc} Tm(Tm'(Tm''J)) & \xrightarrow{\mu_{m,m',Tm''J}} & T(m \cdot m')(Tm''J) \\ {\scriptstyle Tm\mu_{m',m'',J}}\downarrow & & \downarrow{\scriptstyle \mu_{mm',m'',J}} \\ Tm(T(m' \cdot m'')J) & \xrightarrow{\mu_{m,m'm'',J}} & T(m \cdot m' \cdot m'')J \end{array}$$

Graded comonads are dually defined (i.e., as a graded monad on $\mathbb{C}^{op}$).

By mimicking the construction of Kleisli categories, we can construct an $M$-graded category $\mathbb{C}_T$ (we call it the Kleisli $M$-graded category of $T$) from a category $\mathbb{C}$ with an $M$-graded monad $T$ on $\mathbb{C}$.[6]

- $\mathbf{Obj}(\mathbb{C}_T) \triangleq \mathbf{Obj}(\mathbb{C})$ and $\mathbb{C}_T(X, Y)(m) \triangleq \mathbb{C}(X, TmY)$.
- For $f : X \to_m Y$ and $n$ such that $m \leq n$, we define $\uparrow_m^n f \triangleq T(m \leq n)_Y \circ f$.
- Identity and composition are defined by: $\mathrm{id}_X \triangleq \eta_X : X \to_1 X$ and $g \circ f \triangleq \mu_{m,n,Z} \circ Tm\, g \circ f$ for $f : X \to_m Y$ and $g : Y \to_n Z$.

The dual construction is possible. Let $D$ be an $M^{op}$-graded comonad on a category $\mathbb{C}$. We then define $\mathbb{C}_D$ by $\mathbb{C}_D(X, Y)(m) = \mathbb{C}(DmX, Y)$; the rest of data is similar to the case of graded monads. This yields an $M$-graded category $\mathbb{C}_D$.

*Remark 1.* As an aside (included for completeness but not needed in the rest of the paper), graded categories are an instance of *enriched categories*. For the enriching category, we take the presheaf category $[M, \mathbf{Set}]$, together with *Day's convolution product* [10].

[6] Not to be confused with the Kleisli category of graded monads by Fujii et al. [13].

#### 4.1 Homogeneous Coproducts in Graded Categories

We model boolean values and natural numbers by the binary coproduct $1+1$ and the countable coproduct $\coprod_{i\in\mathbb{N}} 1$. We thus define what it means for a graded category to have coproducts. The following definition of binary coproducts easily extends to coproducts of families of objects.

**Definition 2.** Let $\mathbb{C}$ be an $M$-graded category. A *homogeneous binary coproduct* of $X_1, X_2 \in \mathbb{C}$ consists of an object $Z \in \mathbb{C}$ together with injections $\iota_1 \in \mathbb{C}(X_1, Z)(1)$ and $\iota_2 \in \mathbb{C}(X_2, Z)(1)$ such that, for any $m \in M$ and $Y \in \mathbb{C}$, the function $\lambda f\ .\ (f \circ \iota_1, f \circ \iota_2)$ of type $\mathbb{C}(Z,Y)(m) \to \mathbb{C}(X_1,Y)(m) \times \mathbb{C}(X_2,Y)(m)$ is invertible. The inverse is called the *cotupling* and denoted by $[-,-]$. It satisfies the usual law of coproducts ($i = 1, 2$):

$$[f_1, f_2] \circ \iota_i = f_i, \qquad [\iota_1, \iota_2] = \mathrm{id}_Z,$$
$$g \circ [f_1, f_2] = [g \circ f_1, g \circ f_2], \qquad [\uparrow^n_m f_1, \uparrow^n_m f_2] = \uparrow^n_m [f_1, f_2].$$

When homogeneous binary coproducts of any combination of $X_1, X_2 \in \mathbb{C}$ exists, we say that $\mathbb{C}$ has homogeneous binary coproducts.

The difference between homogeneous coproducts and coproducts in ordinary category theory is that the cotupling is restricted to take morphisms with the same grade. A similar constraint is seen in some effect systems, where the typing rule of conditional expressions require each branch to have the same effect.

**Proposition 1.** *Let $\{\iota_i \in \mathbb{C}(X_i, Z)\}_{i\in I}$ be a coproduct of $\{X_i\}_{i\in I}$ in an ordinary category $\mathbb{C}$.*

1. *Suppose that $T$ is an $M$-graded monad on $\mathbb{C}$. Then $\{\eta_Z \circ \iota_i \in \mathbb{C}_T(X_i, Z)(1)\}_{i\in I}$ is a homogeneous coproduct in $\mathbb{C}_T$.*
2. *Suppose that $(D, \varepsilon, \delta)$ is an $M^{op}$-graded comonad on $\mathbb{C}$ such that each $Dm : \mathbb{C} \to \mathbb{C}$ preserves the coproduct $\{\iota_i\}_{i\in I}$. Then $\{\iota_i \circ \varepsilon_I \in \mathbb{C}_D(X_i, Z)(1)\}_{i\in I}$ is a homogeneous coproduct in $\mathbb{C}_D$.*

#### 4.2 Graded Freyd Categories with Countable Coproducts

We now introduce the central categorical structure of the loop language and GHL semantics: *graded Freyd categories* with homogeneous countable coproducts.

**Definition 3.** An $M$-graded Freyd category with homogeneous countable coproducts consists of the following data:

1. A cartesian monoidal category $(\mathbb{V}, 1, \times, l, r, a)$ with countable coproducts such that for all $V \in \mathbb{V}$, the functor $V \times (-) : \mathbb{V} \to \mathbb{V}$ preserves coproducts.
2. An $M$-graded category $\mathbb{C}$ such that $\mathbf{Obj}(\mathbb{C}) = \mathbf{Obj}(\mathbb{V})$ and $\mathbb{C}$ has homogeneous countable coproducts.
3. A function $I_{V,W} : \mathbb{V}(V,W) \to \mathbb{C}(V,W)(1)$ for each $V, W \in \mathbb{C}$. Below we may omit writing subscripts of $I$. The role of this function is to inject pure computations into effectful computations.

4. A function $(*)_{V,X,W,Y} : \mathbb{V}(V,W) \times \mathbb{C}(X,Y)(m) \to \mathbb{C}(V \times X, W \times Y)(m)$ for each $V, W, X, Y \in \mathbb{C}$ and $m \in M$. Below we use it as an infix operator and sometimes omit its subscripts. The role of this function is to combine pure computations and effectful computations in parallel.

The function $I$ and $(*)$ satisfy the following equations:

$$I(\mathrm{id}_X) = \mathrm{id}_X \quad I(g \circ f) = Ig \circ If \quad I(f \times g) = f * Ig \quad \mathrm{id}_V * \mathrm{id}_X = \mathrm{id}_{V*X},$$
$$(g \circ f) * (i \circ j) = (g * i) \circ (f * j) \quad f * {\uparrow}^n_m g = {\uparrow}^n_m (f * g)$$
$$f \circ I(l_X) = I(l_X) \circ (\mathrm{id}_1 * f) \quad I(a_{X',Y',Z'}) \circ ((f \times g) * h) = (f * (g * h)) \circ I(a_{X,Y,Z})$$

These are analogous to the usual Freyd categories axioms. We also require that:

1. For any countable coproduct $\{\iota_i \in \mathbb{V}(X_i, Y)\}_{i \in A}$, $\{I(\iota_i) \in \mathbb{C}(X_i, Y)(1)\}_{i \in A}$ is a homogeneous countable coproduct.
2. For any homogeneous countable coproduct $\{\iota_i \in \mathbb{C}(X_i, Y)(1)\}_{i \in A}$ and $V \in \mathbb{V}$, $\{\mathrm{id}_V * \iota_i \in \mathbb{C}(V \times X_i, V \times Y)(1)\}_{i \in A}$ is a homogeneous countable coproduct.

We denote an $M$-graded Freyd category with countable coproducts by the tuple $(\mathbb{V}, 1, \times, \mathbb{C}, I, (*))$ capturing the main details of the cartesian monoidal structure of $\mathbb{V}$, the base category $\mathbb{C}$, the lifting function $I$ and the action $(*)$.

If the grading pomonoid $M$ is trivial, $\mathbb{C}$ becomes an ordinary category with countable coproducts. We therefore simply call it a Freyd category with countable coproducts. This is the same as a *distributive Freyd category* in the sense introduced by Power [46] and Staton [51]. We will use non-graded Freyd categories to give a semantics of the loop language in Section 4.3. An advantage of Freyd categories is that they encompasses a broad class of models of computations, not limited to those arising from monads. A recent such example is Staton's category of *s-finite kernels* [52][7].

We could give an alternative abstract definition of $M$-graded Freyd category using 2-categorical language: a graded Freyd category is an equivariant morphism in the category of actions from a cartesian category to $M$-graded categories. The full detail of this formulation will be discussed elsewhere.

A Freyd category typically arises from a strong monad on a cartesian category [47]. We give here a graded analogue of this fact. First, we recall the notion of *strength* for graded monads [24, Definition 2.5]. Let $(\mathbb{C}, 1, \times)$ be a cartesian monoidal category. A *strong* $M$-graded monad is a pair of an $M$-graded monad $(T, \eta, \mu)$ and a natural transformation $\mathrm{st}_{I,J,m} \in \mathbb{C}(I \times TmJ, Tm(I \times J))$ satisfying graded versions of the four coherence laws in [34, Definition 3.2]. We dually define a *costrong* $M$-graded comonad $(D, \varepsilon, \delta, \mathrm{cs})$ to be the $M$-graded comonad equipped with the *costrength* $\mathrm{cs}_{I,J,m} \in \mathbb{C}(Dm(I \times J), I \times DmJ)$.

**Proposition 2.** *Let* $(\mathbb{C}, 1, \times)$ *be a cartesian monoidal category.*

1. *Let* $(T, \eta, \mu, \mathrm{st})$ *be a strong* $M$*-graded monad on* $\mathbb{C}$*. The Kleisli* $M$*-graded category* $\mathbb{C}_T$*, together with* $If = \eta_W \circ f$ *and* $f * g = \mathrm{st}_{W,Y} \circ (f \times g)$ *forms an* $M$*-graded Freyd category with homogeneous countable coproducts.*

---
[7] It is not known whether the category of s-finite kernels is a Kleisli category.

*2. Let* $(D, \varepsilon, \delta, \mathrm{cs})$ *be a costrong* $M^{op}$*-graded comonad on* $\mathbb{C}$ *such that each* $Dm$ *preserves countable coproducts. Then the coKleisli* $M$*-graded category* $\mathbb{C}_D$ *together with* $If = f \circ \varepsilon_V$ *and* $f * g = (f \times g) \circ \mathrm{cs}_{V,X}$ *forms an* $M$*-graded Freyd category with homogeneous countable coproducts.*

We often use the following '$\mathsf{ext}$' operation to structure interpretations of programs and GHL derivations. Let $\delta_X \in \mathbb{V}(X, X \times X)$ be the diagonal morphism. Then $\mathsf{ext} : \mathbb{C}(X, Y)(m) \to \mathbb{C}(X, X \times Y)(m)$ is defined as $\mathsf{ext}(f) = (X * f) \circ I\delta_X$. When viewing $X$ as a set of environments, $\mathsf{ext}(f)$ may be seen as executing an effectful procedure $f$ under an environment, then extending the environment with the return value of $f$. In a non-graded setting, the definition of $\mathsf{ext}$ is analogous.

#### 4.3 Semantics of The Loop Language in Freyd Categories

Towards the semantics of GHL, we first give a more standard, non-graded categorical semantics of the loop language. We first prepare the following data.

- ⋆ A Freyd category $(\mathbb{V}, 1, \times, \mathbb{C}, I, *)$ with countable coproducts.
- ⋆ A coproduct $\{\mathsf{tt}, \mathsf{ff} \in \mathbb{V}(1, \mathsf{Bool})\}$ of 1 and 1 in $\mathbb{V}$.
- ⋆ A coproduct $\{\lfloor k \rfloor \in \mathbb{V}(1, \mathsf{Nat})\}_{k \in \mathbb{N}}$ of $\mathbb{N}$-many 1s in $\mathbb{V}$.
- ⋆ An interpretation $[\![-]\!]$ of $\Sigma$ in $\mathbb{V}$ such that

$$[\![\texttt{bool}]\!] = \mathsf{Bool} \qquad [\![\texttt{tt}]\!] = \mathsf{tt} \in \mathbb{V}(1, \mathsf{Bool}) \qquad [\![\texttt{ff}]\!] = \mathsf{ff} \in \mathbb{V}(1, \mathsf{Bool})$$
$$[\![\texttt{nat}]\!] = \mathsf{Nat} \qquad [\![\lceil k \rceil]\!] = \lfloor k \rfloor \in \mathbb{V}(1, \mathsf{Nat}).$$

For convenience, we let $\mathsf{M} \triangleq [\![\Gamma_\mathsf{M}]\!]$ (Section 3.1), i.e., all relevant (mutable) program variables are in scope, and write $\pi_v \in \mathbb{V}(\mathsf{M}, [\![\Gamma_\mathsf{M}(v)]\!])$ for the projection morphism associated to a program variable $v \in \Gamma_\mathsf{M}$.

Pure expressions are interpreted as $\mathbb{V}$-morphisms and impure commands and procedures are interpreted as $\mathbb{C}$-morphisms, of the form:

- ⋆ (expressions) A morphism $[\![e]\!] \in \mathbb{V}(\mathsf{M}, [\![s]\!])$ for all $e \in \mathbf{Exp}_\Sigma(\Gamma_\mathsf{M}, s)$; see Section 3.1.
- ⋆ (commands) A morphism $[\![c]\!] \in \mathbb{C}(\mathsf{M}, 1)$ for each $c \in \mathbf{CExp}$.
- ⋆ (procedures) A morphism $[\![p]\!] \in \mathbb{C}(\mathsf{M}, [\![s]\!])$ for each $s \in S$ and $p \in \mathbf{PExp}_s$.

For the interpretation of programs, we first define some auxiliary morphisms. For all $v \in \Gamma_\mathsf{M}$, let $\mathsf{upd}_v \in \mathbb{V}(\mathsf{M} \times [\![\Gamma_\mathsf{M}(v)]\!], \mathsf{M})$ to be the unique morphism (capturing memory updates) satisfying $\pi_v \circ \mathsf{upd}_v = \pi_2$ and $\pi_w \circ \mathsf{upd}_v = \pi_w \circ \pi_1$ for any $w \in \Gamma_\mathsf{M}$ such that $v \neq w$. We define $\mathsf{sub}(v, e) \in \mathbb{V}(\mathsf{M}, \mathsf{M})$ by $\mathsf{sub}(v, e) \triangleq \mathsf{upd}_v \circ \langle \mathrm{id}_\mathsf{M}, [\![e]\!] \rangle$, which updates the memory configuration at variable $v$ with the value of $e$.

For the interpretation of conditional and loop commands, we need coproducts over $\mathsf{M}$. Since $\mathbb{V}$ is distributive, we can form a binary coproduct $\mathsf{M} \times \mathsf{Bool}$ and a countable coproduct $\mathsf{M} \times \mathsf{Nat}$ with injections respectively defined as $(\forall k \in \mathbb{N})$:

$$\mathsf{tm} \triangleq \langle \mathrm{id}_\mathsf{M}, \mathsf{tt} \circ !_\mathsf{M} \rangle \in \mathbb{V}(\mathsf{M}, \mathsf{M} \times \mathsf{Bool}) \quad [k] \triangleq \langle \mathrm{id}_\mathsf{M}, \lfloor k \rfloor \circ !_\mathsf{M} \rangle \in \mathbb{V}(\mathsf{M}, \mathsf{M} \times \mathsf{Nat})$$
$$\mathsf{fm} \triangleq \langle \mathrm{id}_\mathsf{M}, \mathsf{ff} \circ !_\mathsf{M} \rangle \in \mathbb{V}(\mathsf{M}, \mathsf{M} \times \mathsf{Bool})$$

By Condition 1 of Definition 3, these coproducts are mapped to coproducts in $\mathbb{C}$ with injections:

$$\{I(\mathsf{tm}), I(\mathsf{fm}) \in \mathbb{C}(\mathsf{M}, \mathsf{M} \times \mathsf{Bool})\}, \qquad \{I([k]) \in \mathbb{C}(\mathsf{M}, \mathsf{M} \times \mathsf{Nat}) \mid k \in \mathbb{N}\}.$$

The cotuplings of these coproducts (written $[f, g]$ and $[f^{(k)}]_{k\in\mathbb{N}}$ respectively) are used next to interpret conditionals and loops.
We interpret a program $P$ of the loop language as a morphism $[\![P]\!] \in \mathbb{C}(\mathsf{M}, \mathsf{M})$:

$$\begin{aligned}
[\![P; P']\!] &= [\![P']\!] \circ [\![P]\!] & [\![\mathtt{skip}]\!] &= \mathrm{id}_{\mathsf{M}} \\
[\![\mathtt{do}\, v \leftarrow p]\!] &= I(\mathsf{upd}_v) \circ \mathsf{ext}[\![p]\!] & [\![\mathtt{do}\, c]\!] &= I(\pi_1) \circ \mathsf{ext}[\![c]\!] \\
[\![v := e]\!] &= I(\mathsf{sub}(v, e)) \\
[\![\mathtt{if}\, e_b\, \mathtt{then}\, P\, \mathtt{else}\, P']\!] &= [\, [\![P]\!], [\![P']\!]\,] \circ \mathsf{ext}(I[\![e_b]\!]) \\
[\![\mathtt{loop}\, e_n\, \mathtt{do}\, P]\!] &= [\, [\![P]\!]^{(k)}\,]_{k\in\mathbb{N}} \circ \mathsf{ext}(I[\![e_n]\!])
\end{aligned}$$

Thus, the semantics of $\mathtt{loop}\, e_n\, \mathtt{do}\, P$ is such that, if the expression $e_n$ evaluates to some natural number $\lceil k \rceil$ then $\mathtt{loop}\, e_n \mathtt{do} P$ is equivalent to the $k$-times sequential composition of $P$.

## 5 Modelling Graded Hoare Logic

We now define the categorical model of GHL, building on the non-graded Freyd semantics of Section 4.3. Section 5.1 first models the base assertion logic, for which we use fibrations, giving an overview of the necessary mathematical machinery for completeness. Section 5.2 then defines the semantics of GHL and Section 5.3 instantiates it for the examples discussed previously in Section 3.

### 5.1 Interpretation of the Assertion Logic using Fibrations

Our assertion logic (Section 3) has logical connectives of finite conjunctions, countable disjunctions, existential quantification and an equality predicate. A suitable categorical model for this fragment of first-order logic is offered by a *coherent fibration* [22, Def. 4.2.1], extended with countable joins in each fibre. We recap various key definitions and terminology due to Jacobs' textbook [22].

In the following, let $\mathbb{P}$ and $\mathbb{V}$ be categories and $p : \mathbb{P} \to \mathbb{V}$ a functor.

We can regard functor $p$ as attaching *predicates* to each object in $\mathbb{V}$. When $p\psi = X$, we regard $\psi \in \mathbb{P}$ as a predicate over $X \in \mathbb{V}$. When $f \in \mathbb{P}(\psi, \phi)$ is a morphism, we regard this as saying that $pf$ maps elements satisfying $\psi$ to those satisfying $\phi$ in $\mathbb{V}$. Parallel to this view of functors assigning predicates is the notion that entities in $\mathbb{P}$ are 'above' those in $\mathbb{V}$ when they are mapped to by $p$.

**Definition 4 ('Aboveness').** An object $\psi \in \mathbb{P}$ is said to be *above* an object $X \in \mathbb{V}$ if $p\psi = X$. Similarly, a morphism[8] $\dot{f} \in \mathbb{P}(\psi, \phi)$ is said to be *above* a morphism $f$ in $\mathbb{V}$ if $p\dot{f} = f \in \mathbb{V}(p\psi, p\phi)$. A morphism in $\mathbb{P}$ is *vertical* if it is above an identity morphism. Given $\psi, \phi \in \mathbb{P}$ and $f \in \mathbb{V}(p\psi, p\phi)$, then we denote the *set of all morphisms in* $\mathbb{P}$ *above* $f$ as $\mathbb{P}_f(\psi, \phi) = \{\dot{f} \in \mathbb{P}(\psi, \phi) \mid p\dot{f} = f\}$.

[8] The dot notation here introduces a new name and should not be understood as applying some mathematical operator on $f$.

**Definition 5 (Fibre category).** A *fibre category* over $X \in \mathbb{V}$ is a subcategory of $\mathbb{P}$ consisting of objects above $X$ and morphisms above $\mathrm{id}_X$. This subcategory is denoted by $\mathbb{P}_X$, and thus the homsets of $\mathbb{P}_X$ are $\mathbb{P}_X(\psi, \phi) = \mathbb{P}_{\mathrm{id}_X}(\psi, \phi)$.

We are ready to recall the central concept in fibrations: *cartesian morphisms.*

**Definition 6 (Cartesian morphism).** A morphism $\dot{f} \in \mathbb{P}(\psi, \phi)$ is *cartesian* if for any $\alpha \in \mathbb{P}$ and $g \in \mathbb{V}(p\alpha, p\psi)$, the post-composition of $\dot{f}$ in $\mathbb{P}$, regarded as a function of type $\dot{f} \circ - : \mathbb{P}_g(\alpha, \psi) \to \mathbb{P}_{g \circ p\dot{f}}(\alpha, \phi)$, is a bijection. This amounts to the following *universal property* of cartesian morphism: for any $\dot{h} \in \mathbb{P}(\alpha, \phi)$ above $g \circ pf$, there exists a unique morphism $\dot{g} \in \mathbb{P}(\alpha, \psi)$ above $g$ such that $\dot{h} = \dot{f} \circ \dot{g}$. Intuitively, $\dot{f}$ represents the situation where $\psi$ is a *pullback* or *inverse image* of $\phi$ along $p\dot{f}$, and the universal property corresponds to that of pullback.

**Definition 7 (Fibration).** Finally, a functor $p : \mathbb{P} \to \mathbb{V}$ is a *fibration* if for any $\psi \in \mathbb{P}$, $X \in \mathbb{V}$, and $f \in \mathbb{V}(X, p\psi)$, there exists an object $\phi \in \mathbb{P}$ and a cartesian morphism $\dot{f} \in \mathbb{P}(\phi, \psi)$ above $f$, called the *cartesian lifting* of $f$ with $\psi$. We say that a fibration $p : \mathbb{P} \to \mathbb{V}$ is *posetal* if each $\mathbb{P}_X$ is a poset, corresponding to the implicational order between predicates. When $\psi \le \phi$ holds in $\mathbb{P}_X$, we denote the corresponding vertical morphism in $\mathbb{P}$ as $\psi \nearrow \phi$.

Posetal fibrations are always faithful. The cartesian lifting of $f \in \mathbb{V}(X, p\psi)$ with $\psi$ uniquely exists. We thus write it by $\overline{f}\psi$, and its domain by $f^*\psi$. It can be easily shown that for any morphism $f \in \mathbb{V}(X, Y)$ in $\mathbb{V}$, the assignment $\psi \in \mathbb{P}_Y \mapsto f^*\psi \in \mathbb{P}_X$ extends to a monotone function $f^* : \mathbb{P}_Y \to \mathbb{P}_X$. We call it the *reindexing function* (along $f$). Furthermore, the assignment $f \mapsto f^*$ satisfies the (contravariant) functoriality: $\mathrm{id}_X^* = \mathrm{id}_{\mathbb{P}_X}$ and $(g \circ f)^* = f^* \circ g^*$. A fibration is a *bifibration* if each reindexing function $f^* : \mathbb{P}_Y \to \mathbb{P}_X$ for $f \in \mathbb{V}(X, Y)$ has a left adjoint, denoted by $f_* : \mathbb{P}_X \to \mathbb{P}_Y$. $f_*\psi$ is always associated with a morphism $\underline{f}\psi : f_*\psi \to \psi$ above $f$, and this is called the *opcartesian lifting* of $f$ with $\psi$. For the universal property of the opcartesian lifting, see Jacobs [22, Def. 9.1.1].

*Fibrations for our Assertion Logic* It is widely known that *coherent fibrations* are suitable for interpreting the $\wedge, \vee, \exists, =$-fragment of first-order logic (see [22, Chapter 4, Def. 4.2.1]). Based on this fact, we introduce a class of fibrations that are suitable for our assertion logic—due to the countable joins of the assertion logic we modify the definition of coherent fibration accordingly.

**Definition 8.** A *fibration for assertion logic* over $\mathbb{V}$ is a posetal fibration $p : \mathbb{P} \to \mathbb{V}$ for cartesian $\mathbb{V}$ with distributive countable coproducts, such that:

1. Each fibre poset $\mathbb{P}_X$ is a distributive lattice with finite meets $\top_X, \wedge$ and countable joins $\bot_X, \vee$.
2. Each reindexing function $f^*$ preserves finite meets and countable joins.
3. The reindexing function $c_{X,Y}^*$ along the contraction $c_{X,Y} \triangleq \langle \pi_1, \pi_2, \pi_2 \rangle \in \mathbb{V}(X \times Y, X \times Y \times Y)$ has a left adjoint $\mathrm{Eq}_{X,Y} \dashv c_{X,Y}^*$. This satisfies *Beck-Chevalley condition* and *Frobenius property*; we refer to [22, Definition 3.4.1].

4. The reindexing function $w^*_{X,Y}$ along the weakening $w_{X,Y} \triangleq \pi_1 \in \mathbb{V}(X \times Y, X)$ has a left adjoint $\exists_{X,Y} \dashv w^*_{X,Y}$. This satisfies *Beck-Chevalley condition* and *Frobenius property*; we refer [22, Definition 1.9.1, 1.9.12].

This is almost the same as the definition of coherent fibrations [22, Definition 4.2.1]; the difference is that 1) the base category $\mathbb{V}$ has countable coproducts 2) we require each fibre to be a poset; this makes object equalities hold on-the-nose, and 3) we require each fibre to have countable joins. They will be combined with countable coproducts of $\mathbb{V}$ to equip $\mathbb{P}$ with a countable coproduct [22].

*Example 6.* A typical example of a fibration for assertion logic is the subobject fibration $p^{\mathbf{Set}} : \mathbf{Pred} \to \mathbf{Set}$; the category **Pred** has objects pairs $(X, \psi)$ of sets such that $\psi \subseteq X$, and morphisms of type $(X, \psi) \to (Y, \phi)$ as functions $f : X \to Y$ such that $f(\psi) \subseteq \phi$. The functor $p$ sends $(X, \psi)$ to $X$ and $f$ to itself. More examples can be found in the work of Jacobs [22, Section 4].

For a parallel pair of morphisms $f, g \in \mathbb{V}(X, Y)$, we define the equality predicate $\mathrm{Eq}(f, g)$ above $X$ to be $\langle \mathrm{id}_X, f, g\rangle^* \mathrm{Eq}_{X,Y}(\top_{X \times Y})$ [22, Notation 3.4.2]. Intuitively, $\mathrm{Eq}(f, g)$ corresponds to the predicate $\{x \in X \mid f(x) = g(x)\}$. In this paper, we will use some facts about the equality predicate shown by Jacobs [22, Proposition 3.4.6, Lemma 3.4.5, Notation 3.4.2, Example 4.3.7].

**The Semantics of Assertion Logic** We move to the semantics of our assertion logic in a fibration $p : \mathbb{P} \to \mathbb{V}$ for assertion logic. The basic idea is to interpret a formula $\psi \in \mathbf{Fml}_{\Sigma_l}(\Gamma)$ as an object in $\mathbb{P}_{[\![\Gamma]\!]}$, and an entailment $\Gamma \mid \psi \vdash \phi$ as the order relation $[\![\psi]\!] \leq [\![\phi]\!]$ in $\mathbb{P}_{[\![\Gamma]\!]}$. The semantics is given by the following interpretation of the data specifying the assertion logic (given in Section 3.3):

⋆ A fibration $p : \mathbb{P} \to \mathbb{V}$ for assertion logic.
⋆ An interpretation $[\![-]\!]$ of $\Sigma_l$ in $\mathbb{P}$ that coincides with the one $[\![-]\!]$ of $\Sigma$ in $\mathbb{V}$.
⋆ An object $[\![P]\!] \in \mathbb{P}_{[\![par(P)]\!]}$ for each atomic proposition $P \in P_l$ (recall *par* assigns input sorts to atomic propositions in $P_l$, parameterising the logic).
⋆ We require that for any $\Gamma \in \mathbf{Ctx}_{\Sigma_l}$ and $(\psi, \phi) \in \mathbf{Axiom}(\Gamma)$, $[\![\psi]\!] \leq [\![\phi]\!]$ holds in $\mathbb{P}_{[\![\Gamma]\!]}$. This expresses an implicational axiom in the coherent logic.

The interpretation $[\![\psi]\!]$ of $\psi \in \mathbf{Fml}_{\Sigma_l}(\Gamma)$ is inductively defined as a $\mathbb{P}_{[\![\Gamma]\!]}$-object:

$$[\![P(t_1, \cdots, t_n)]\!] = \langle [\![t_1]\!], \cdots, [\![t_n]\!]\rangle^* [\![P]\!] \qquad [\![t = u]\!] = \mathrm{Eq}([\![t]\!], [\![u]\!])$$
$$[\![\bigwedge \psi_i]\!] = \bigwedge [\![\psi_i]\!] \qquad [\![\bigvee \psi_i]\!] = \bigvee [\![\psi_i]\!] \qquad [\![\exists x : s \;.\; \psi]\!] = \exists_{[\![\Gamma]\!], [\![s]\!]} [\![\psi]\!]$$

### 5.2 Interpretation of Graded Hoare Logic

We finally introduce the semantics of Graded Hoare logic. This semantics interprets derivations of GHL judgements $\vdash_m \{\psi\}\ P\ \{\phi\}$ as $m$-graded morphisms in a graded category. Moreover, it is built *above* the interpretation $[\![P]\!] \in \mathbb{C}(\mathsf{M}, \mathsf{M})$ of the program $P$ in the non-graded semantics introduced in Section 4.3. The underlying structure is given as a combination of a fibration for the assertion logic and a graded category over $\mathbb{C}$, as depicted in (1) (Section 2, p. 237).

**Definition 9.** A *GHL structure* over a Freyd category $(\mathbb{V}, 1, \times, \mathbb{C}, I, *)$ with countable coproducts and a fibration $p : \mathbb{P} \to \mathbb{V}$ for assertion logic comprises:

1. An $M$-graded Freyd category $(\mathbb{P}, \dot{1}, \dot{\times}, \mathbb{E}, \dot{I}, \circledast)$ with homogeneous countable coproducts.
2. A function $q_{\psi,\phi,m} : \mathbb{E}(\psi, \phi)(m) \to \mathbb{C}(p\psi, p\phi)$ (subscripts may be omitted), which maps to the base denotational model, erasing assertions and grades.

The above data satisfy the following properties:

1. That $q$ behaves 'functorialy' preserving structure from $\mathbb{E}$ to $\mathbb{V}$:

$$q(\mathrm{id}_\phi) = \mathrm{id}_{p\phi}, \quad q(g \circ f) = qg \circ qf, \quad q_{\psi,\phi,n}(\uparrow^n_m f) = q_{\psi,\phi,m} f$$
$$q(\dot{I} f) = I(pf), \quad q(f \circledast g) = pf * qg$$

2. For any homogeneous countable coproduct $\{\iota_i \in \mathbb{E}(\psi_i, \phi)(1)\}_{i \in \Lambda}$, $\{q\iota_i \in \mathbb{C}(p\psi_i, p\phi)\}_{i\in\Lambda}$ is a countable coproduct.
3. (Ex falso quodlibet) $q_{\perp_X,\phi,m} : \mathbb{E}(\perp_X, \phi)(m) \to \mathbb{C}(X, p\phi)$ is a bijection.

The last statement asserts that if the precondition is the least element $\perp_X$ in the fibre over $X \in \mathbb{V}$, which represents the false assertion, we trivially conclude any postcondition $\phi$ and grading $m$ for any morphisms of type $X \to p\phi$ in $\mathbb{C}$.

The semantics of GHL then requires a graded Freyd category with countable coproducts, and morphisms in the graded category guaranteeing a sound model of the effectful primitives (commands/procedures), captured by the data:

- ⋆ A GHL structure $(\mathbb{P}, \dot{1}, \dot{\times}, \mathbb{E}, \dot{I}, \circledast, q)$ over the Freyd category $(\mathbb{V}, 1, \times, \mathbb{C}, I, *)$ with countable coproducts and the fibration $p : \mathbb{P} \to \mathbb{V}$ for assertion logic.
- ⋆ For each $c \in C_{\mathsf{c}}(\psi, m)$ a morphism $\langle c\rangle \in \mathbb{E}([\![\psi]\!], \dot{1})(m)$ such that $q\langle c\rangle = [\![c]\!]$.
- ⋆ For each $p \in C^s_{\mathsf{p}}(\psi, m, \phi)$ a morphism $\langle p\rangle \in \mathbb{E}([\![\psi]\!], [\![\phi]\!])(m)$ such that $q\langle p\rangle = [\![p]\!]$.

where $[\![c]\!]$, $[\![p]\!]$ and later $[\![e]\!]$ are from the underlying non-graded model (Sec. 4.3).

We interpret a derivation of GHL judgement $\vdash_m \{\phi\}\ P\ \{\psi\}$ as a morphism

$$[\![\vdash_m \{\phi\}\ P\ \{\psi\}]\!] \in \mathbb{E}([\![\phi]\!], [\![\psi]\!])(m) \ \text{ such that } \ q_{[\![\phi]\!],[\![\psi]\!],m}[\![\vdash_m \{\phi\}\ P\ \{\psi\}]\!] = [\![P]\!].$$

The constraint on the right is guaranteed by the soundness of the interpretation (Theorem 1). From the functor-as-refinement viewpoint [32], the interpretation $[\![\vdash_m \{\phi\}\ P\ \{\psi\}]\!]$ witnesses that $[\![P]\!]$ respects refinements $\phi$ and $\psi$ of $\mathsf{M}$, and additionally it witnesses the grade of $[\![P]\!]$ being $m$. We first cover the simpler cases of the interpretation of GHL derivations:

$$[\![\vdash_1 \{\psi\}\ \mathtt{skip}\ \{\psi\}]\!] = \mathrm{id}_{[\![\psi]\!]}$$
$$[\![\vdash_{m_1\cdot m_2} \{\psi\}\ P_1 ; P_2\ \{\theta\}]\!] = [\![\vdash_{m_2} \{\psi_1\}\ P_2\ \{\theta\}]\!] \circ [\![\vdash_{m_1} \{\psi\}\ P_1\ \{\psi_1\}]\!]$$
$$[\![\vdash_1 \{\psi[e/v]\}\ v := e\ \{\psi\}]\!] = \dot{I}(\overline{\mathsf{sub}(v, e)}[\![\psi]\!])$$
$$[\![\vdash_m \{\psi\}\ \mathtt{do}\, c\ \{\psi\}]\!] = \dot{I}(\pi_1) \circ \mathsf{ext}\langle c\rangle$$
$$[\![\vdash_m \{\psi\}\ \mathtt{do}\, v \leftarrow p\ \{(\exists v\ .\ \psi) \wedge \phi\}]\!] = \dot{I}(\underline{\mathsf{upd}_v}([\![\psi]\!]\ \dot{\times}\ [\![\phi]\!])) \circ \mathsf{ext}\langle p\rangle$$
$$[\![\vdash_{m'} \{\psi'\}\ P\ \{\phi'\}]\!] = \dot{I}([\![\phi]\!] \nearrow [\![\phi']\!]) \circ \uparrow^{m'}_m [\![\vdash_m \{\psi\}\ P\ \{\phi\}]\!] \circ \dot{I}([\![\psi']\!] \nearrow [\![\psi]\!])$$

The morphisms with upper and lower lines are cartesian liftings and op-cartesian liftings in the fibration $p : \mathbb{P} \to \mathbb{V}$ of the assertion logic. The codomain of the interpretation of the procedure call $\mathtt{do}\, v \leftarrow p$ is equal to $[\![(\exists v \,.\, \psi) \wedge \phi]\!]$.

The above interpretations largely follow the form of the underlying model of Section 4.3, with the additional information and underlying categorical machinery for grades and assertions here; we now map to $\mathbb{E}$. The interpretation of conditional and loop commands requires some more reasoning.

*Conditionals* Let $p_1, p_2$ be the interpretations of each branch of the conditional command:

$$p_1 = [\![\vdash_m \{\psi \wedge e_b = \mathtt{tt}\}\ P_1\ \{\phi\}]\!] \in \mathbb{E}([\![\psi \wedge e_b = \mathtt{tt}]\!], [\![\phi]\!])(m)$$
$$p_2 = [\![\vdash_m \{\psi \wedge e_b = \mathtt{ff}\}\ P_2\ \{\phi\}]\!] \in \mathbb{E}([\![\psi \wedge e_b = \mathtt{ff}]\!], [\![\phi]\!])(m)$$

We consider the cocartesian lifting $\underline{\langle \mathrm{id}_{\mathsf{M}}, [\![e_b]\!]\rangle}[\![\psi]\!] : [\![\psi]\!] \to \langle \mathrm{id}_{\mathsf{M}}, [\![e_b]\!]\rangle_* [\![\psi]\!]$. We name its codomain $\mathsf{Im}$. Next, cartesian morphisms $\overline{\mathsf{tm}}(\mathsf{Im}) : \mathsf{tm}^*\mathsf{Im} \to \mathsf{Im}$ and $\overline{\mathsf{fm}}(\mathsf{Im}) : \mathsf{fm}^*\mathsf{Im} \to \mathsf{Im}$ in $\mathbb{P}$ are above the coproduct $(\mathsf{M} \times \mathsf{Bool}, \mathsf{tm}, \mathsf{fm})$ in $\mathbb{V}$. Then the interpretations of the preconditions of $P_1, P_2$ are inverse images of $\mathsf{Im}$ along $\mathsf{tm}, \mathsf{fm} : \mathsf{M} \to \mathsf{M} \times \mathsf{Bool}$:

**Lemma 1.** $[\![\psi \wedge e_b = \mathtt{tt}]\!] = \mathsf{tm}^*\mathsf{Im}$ *and* $[\![\psi \wedge e_b = \mathtt{ff}]\!] = \mathsf{fm}^*\mathsf{Im}$.

The side condition of the conditional rule ensures that $(\mathsf{Im}, \overline{\mathsf{tm}}(\mathsf{Im}), \overline{\mathsf{fm}}(\mathsf{Im}))$ is a coproduct in $\mathbb{P}$:

**Lemma 2.** $\Gamma_{\mathsf{M}} \mid \psi \vdash e_b = \mathtt{tt} \vee e_b = \mathtt{ff}$ *implies* $\mathsf{Im} = \mathsf{tm}_*\mathsf{tm}^*\mathsf{Im} \vee \mathsf{fm}_*\mathsf{fm}^*\mathsf{Im}$.

Therefore the image of the coproduct $(\mathsf{Im}, \overline{\mathsf{tm}}(\mathsf{Im}), \overline{\mathsf{fm}}(\mathsf{Im}))$ by $\dot{I}$ yields a homogeneous coproduct in $\mathbb{E}$. We take the cotupling $[p_1, p_2] \in \mathbb{E}(\mathsf{Im}, [\![\phi]\!])(m)$ with respect to this homogeneous coproduct. We finally define the interpretation of the conditional rule to be the following composite:

$$[\![\vdash_m \{\psi\}\mathtt{if}\, e_b\, \mathtt{then}\, P_1\, \mathtt{else}\, P_2\{\phi\}]\!] = [p_1, p_2] \circ \dot{I}(\underline{\langle \mathrm{id}_{\mathsf{M}}, [\![e_b]\!]\rangle}[\![\psi]\!]) \in \mathbb{E}([\![\psi]\!], [\![\phi]\!])(m).$$

*Loops* Fix $N \in \mathbb{N}$, and suppose that $\vdash_m \{\psi_{i+1}\}\, P\, \{\psi_i\}$ is derivable in the graded Hoare logic for each $0 \le i < N$. Let $p_i \in \mathbb{E}([\![\psi_{i+1}]\!], [\![\psi_i]\!])(m)$ be the interpretation $[\![\vdash_m \{\psi_{i+1}\}\ P_i\ \{\psi_i\}]\!]$. We then define a countable family of morphisms (we use here ex falso quodlibet):

$$b_i = \begin{cases} q^{-1}_{\perp_{\mathsf{M}}, [\![\psi_0]\!], m^N}([\![P]\!]^{(i)}) \in \mathbb{E}(\perp_{\mathsf{M}}, [\![\psi_0]\!])(m^N) & (i \neq N) \\ p_0 \circ \cdots \circ p_N \in \mathbb{E}([\![\psi_N]\!], [\![\psi_0]\!])(m^N) & (i = N) \end{cases}$$

Let $\theta_i \triangleq \mathrm{cod}(b_i)$. Then $\coprod_{i \in \mathbb{N}} \theta_i = \bigvee_{i \in \mathbb{N}} [i]_* \theta_i = [N]_* [\![\psi_N]\!]$ because $[i]_* \theta_i$ is either $\perp_{\mathsf{M} \times \mathsf{Nat}}$ or $[N]_* [\![\psi_N]\!]$. We then send the coproduct $\theta_i \to \coprod_{i \in \mathbb{N}} \theta_i$ by $\dot{I}$ and obtain a homogeneous coproduct in $\mathbb{E}$. By taking the cotupling of all $b_i$ with this homogeneous coproduct, we obtain a morphism $[b_i]_{i \in \mathbb{N}} \in \mathbb{E}([N]_* [\![\psi_N]\!], [\![\psi_0]\!])(m^N)$.

**Lemma 3.** $\Gamma_{\mathsf{M}} \mid \psi_N \vdash e_n = \lceil N \rceil$ *implies* $\langle \mathrm{id}_{\mathsf{M}}, [\![e_N]\!]\rangle_* [\![\psi_N]\!] = [N]_* [\![\psi_N]\!]$.

We then define $[\![\vdash_{m^N} \{\psi_N\}\ \mathtt{loop}\, e_n\ \mathtt{do}\ P\ \{\psi_0\}]\!] = [b_i]_{i\in\mathbb{N}} \circ \dot{I}(\underline{\langle \mathrm{id}_{\mathsf{M}}, [\![e_n]\!]\rangle}[\![\psi_N]\!])$.

**Theorem 1 (Soundness of GHL).** *For any derivation of a GHL judgement $\vdash_m \{\phi\}\ P\ \{\psi\}$, we have $q_{[\![\phi]\!],[\![\psi]\!],m}[\![\vdash_m \{\phi\}\ P\ \{\psi\}]\!] = [\![P]\!]$.*

### 5.3 Instances of Graded Hoare Logic

We first present a construction of GHL structures from graded monad liftings, which are a graded version of the concept of *monad lifting* [11,19,26].

**Definition 10.** [Graded Liftings of Monads] Consider two cartesian categories $\mathbb{E}$ and $\mathbb{C}$ and a functor $q\colon \mathbb{E} \to \mathbb{C}$ strictly preserving finite products. We say that a strong $M$-graded monad $(\dot{T}, \dot{\eta}, \dot{\mu}_{m,m'}, \dot{\mathrm{st}}_m)$ on $\mathbb{E}$ is an *$M$-graded lifting* of a strong monad $(T, \eta^T, \mu, \mathrm{st})$ on $\mathbb{C}$ along $q$ if $q \circ \dot{T}m = T \circ q$, $q(\dot{\eta}_\psi) = \eta_{q\psi}$, $q(\dot{\mu}_{m,m',\psi}) = \mu_{q\psi}$, $q(\dot{T}(m_1 \le m_2)_\psi) = \mathrm{id}$, $q(\dot{\mathrm{st}}_{\psi,\phi,m}) = \mathrm{st}_{q\psi,q\phi}$.

**Theorem 2.** *Let $\mathbb{V}$ be cartesian category with distributive countable coproducts, and let $p\colon \mathbb{P} \to \mathbb{V}$ be a fibration for assertion logic. Let $T$ be a strong monad on $\mathbb{V}$ and $\dot{T}$ be an $M$-graded lifting of $T$ along $p$. Then the $M$-graded Freyd category $(\mathbb{P}, 1, \dot{\times}, \mathbb{P}_{\dot{T}}, J, \circledast)$ with homogeneous countable coproducts, together with the function $q_{\psi,\phi,m}\colon \mathbb{P}_{\dot{T}}(\psi,\phi)(m) \to \mathbb{V}_T(p\psi, p\phi)$ defined by $q_{\psi,\phi,m}(f) = pf$ is a GHL structure over $(\mathbb{V}, 1, \times, \mathbb{V}_T, I, *)$ and $p$.*

Before seeing examples, we introduce a notation and fibrations for the assertion logic. Let $p : \mathbb{P} \to \mathbb{V}$ be a fibration for the assertion logic. Below we use the following notation: for $f \in \mathbb{V}(I, J)$ and $\psi \in \mathbb{P}_I$ and $\phi \in \mathbb{P}_J$, by $f : \psi \dot{\to} \phi$ we mean the statement "there exists a morphism $\dot{f} \in \mathbb{P}(\psi, \phi)$ such that $p\dot{f} = f$". Such $\dot{f}$ is unique due to the faithfulness of $p : \mathbb{P} \to \mathbb{V}$.

*Example 7 (Example 4: Union Bound Logic).* To derive the GHL structure suitable for the semantics of the Union Bound Logic discussed in Example 4, we invoke Theorem 2 by letting $p$ be $p^{\mathbf{Set}} : \mathbf{Pred} \to \mathbf{Set}$ (Example 6), $T$ be the subdistribution monad $\mathcal{D}$ and $\dot{T}$ be the strong $(\mathbb{R}_{\ge 0}, \le, 0, +)$-graded lifting $\mathcal{U}$ of $\mathcal{D}$ defined by $\mathcal{U}(\delta)(X, P) \triangleq (\mathcal{D}(X), \{d \mid d(X \setminus P) \le \delta\})$. The induced GHL structure is suitable for the semantics of GHL for Union Bound Logic in Example 4. The soundness of inference rules follow from the GHL structure as we have showed in Section 5.2. To complete the semantics of GHL for the Union Bound Logic, we give the semantics $\langle p\rangle$ of procedures $p \in C^s_{\mathsf{p}}$. Example 4 already gave a semantic condition for these operators:

$$
\begin{aligned}
&C^s_{\mathsf{p}}(\phi, \beta, \psi)\\
&= \{\mathtt{sample}_{\mu,e} \mid \forall s. s \in [\![\phi]\!] \implies \Pr_{s' \leftarrow [\![\mathtt{sample}_{\mu,e}]\!](s)}[s' \in [\![\neg\psi]\!] \le \beta)\}\\
&= \{\mathtt{sample}_{\mu,e} \mid [\![\mathtt{sample}_{\mu,e}]\!] \in \mathbf{Pred}_{\mathcal{U}}([\![\phi]\!], [\![\psi]\!])(\beta)\}
\end{aligned}
$$

For any $\mathtt{sample}_{\mu,e} \in C_p(\phi, \beta, \psi)$, the interpretation $\langle \mathtt{sample}_{\mu,e}\rangle$ is $[\![\mathtt{sample}_{\mu,e}]\!]$.

*Example 8 (Example 3: Program Counter Security).* To derive the GHL structure suitable for GHL with program counter security, we invoke Theorem 2 with:

– The category **ERel** of endorelations defined as follows: an object $(X, R)$ is a pair of $X \in \mathbf{Set}$ and $R \subseteq X \times X$ (i.e. an endorelation $R$ on $X$) and an arrow $f\colon (X, R) \to (Y, S)$ is a function $f\colon X \to Y$ such that $(f \times f)(R) \subseteq S$.
– The fibration for the assertion logic $e : \mathbf{ERel} \to \mathbf{Set}$ given by $(X, R) \mapsto X$ and $f \mapsto f$.
– The writer monad $W_s X = X \times \{\mathtt{tt}, \mathtt{ff}\}^*$ on **Set** with the monoid of bit strings.
– The strong $2^*$-graded lifting of $W_s$ along $e : \mathbf{ERel} \to \mathbf{Set}$, given by $\dot{W}_s\sigma(X, R) = (W_s X, \{((x, \sigma'), (y, \sigma')) \mid (x, y) \in R \wedge \sigma' \le \sigma\})$.

The derived GHL structure is suitable for the semantics of GHL in Example 3. To complete the structure of the logic, we need to interpret two commands $\mathtt{cfTT}, \mathtt{cfFF} \in \mathbf{CExp}$ and set the axioms of commands $C_{\mathsf{c}}$.

First $[\![\mathtt{cfTT}]\!], [\![\mathtt{cfFF}]\!]\colon [\![\mathsf{M}]\!] \to 1$ in $\mathbf{ERel}_{\dot{W}}$ are defined by $[\![\mathtt{cfTT}]\!] \equiv (*, \mathtt{tt})$ and $[\![\mathtt{cfFF}]\!] \equiv (*, \mathtt{ff})$. Finally, we define $C_{\mathsf{c}}$ by (recall $\le$ is prefix ordering of strings):

$$C_{\mathsf{c}}(\psi, \sigma) = \{\mathtt{cfTT} \mid \mathtt{tt} \le \sigma\} \cup \{\mathtt{cfFF} \mid \mathtt{ff} \le \sigma\}.$$

Note, the graded lifting $\dot{W}_s\sigma$ relates only the pair of $(x, \sigma')$ and $(y, \sigma')$ with common strings of control flow. Hence, the derivation of proof tree of this logic forces the target program to have the same control flow under the precondition.

*Example 9 (GHL Structure from the product comonad).* In the category **Set**, the functor $CX \triangleq X \times \mathbb{N}$ forms a coproduct-preserving comonad called the *product comonad*. The right adjoint $I\colon \mathbf{Set} \to \mathbf{Set}_C$ of the coKleisli resolution of $C$ yields a Freyd category with countable coproducts. We next introduce a $(\mathbb{N}, \le, 0\max)$-graded lifting $\dot{C}$ of the comonad $C$ along the fibration $p^{\mathbf{Set}}\colon \mathbf{Pred} \to \mathbf{Set}$. It is defined by $\dot{C}n(X, P) \triangleq (CX, \{(x, m) \in X \times \mathbb{N} \mid x \in P, m \ge n\})$. Similarly, we give an $(\mathbb{N}, \le, 0\max)$-graded Freyd category $(J, \circledast)$ induced by the graded lifting $\dot{C}$. In this way we obtain a GHL structure.

By instantiating GHL with the above GHL structure, we obtain a program logic useful for reasoning about security levels. For example, when program $P_1$ requires security level 3 and $P_2$ requires security level 7, the sequential composition $P_1; P_2$ requires the higher security level 7 ($= \max(3, 7)$).

We give a simple structure for verifying security levels determined by memory access. Fix a function $\mathsf{VarLV}\colon \mathrm{dom}(\Gamma_{\mathsf{M}}) \to \mathbb{N}$ assigning security levels to variables. For any expression $e$, we define its required security level $\mathtt{SecLV}(e) = \sup\{\mathsf{VarLV}(x) \mid x \in \mathrm{FV}(e)\}$. Using this, for each expression $e$ of sort $s \in S$ we introduce a procedure $\mathtt{secr}_e \in \mathbf{PExp}_s$ called *secured expression*. It returns the value of $e$ if the level is high enough, otherwise it returns a meaningless contant:

$$[\![\mathtt{secr}_e]\!](n, \xi) = \text{if } n \ge \mathtt{SecLV}(e) \text{ then } [\![e]\!](\xi) \text{ else a fixed constant } c_s.$$

Secured expressions can be introduced through the following $C_{\mathsf{p}}$:

$$C^s_{\mathsf{p}}(\phi, l, \psi) = \{\mathtt{secr}_e \mid e : s, [\![\mathtt{secr}_e]\!] \in \mathbf{Pred}_C([\![\phi]\!], [\![\psi]\!])(l), \mathsf{SecLV}(e) \le l\}.$$

The pomonoid $(\mathbb{N}, \le, 0, \max)$ in the above can also be replaced with a join semilattice with a least element $(Q, \le, \bot, \vee)$. Thus, GHL can be instantiated to a graded comonadic model of security and its associated reasoning.

### 6 Related Work

Several works have studied abstract semantics of Hoare Logic. Martin et al. [31] give a categorical framework based on traced symmetric monoidal closed categories. They also show that their framework can handle extensions such as separation logic. However their framework does not directly model effects and it cannot accommodate grading as is. Goncharov and Shröder [18] study a Hoare Logic to reason in a generic way about programs with side effects. Their logic and underlying semantics is based on an order-enriched monad and they show a relative completeness result. Similarly, Hasuo [20] studies an abstract weakest precondition semantics based on order-enriched monad. A similar categorical model has also been used by Jacobs [23] to study the Dijkstra monad and the Hoare monad. In the logic by Goncharov and Shröder [18] effects are encapsulated in monadic types, while the weakest precondition semantics by Hasuo [20] and the semantics by Jacobs [23] have no underlying calculus. Moreover, none of them is graded. Maillard et al. [29] study a semantics framework based on the Dijkstra monad for program verification. Their framework enables reasoning about different side effects and it separates specification from computation. Their Dijkstra monad has a flavor of grading but the structure they use is more complex than a pomonoid. Maillard et al. [30] focus on relational program logics for effectful computations. They show how these logics can be derived in a relational dependent type theory, but their logics are not graded.

As we discussed in the introduction, several works have used *grading* structures similar to the one we study in this paper, although often with different names. Katsumata studied monads graded by a pomonoid as a semantic model for effects system [24]. A similar approach has also been studied elsewhere [36,42]. Formal categorical properties of graded monads are pursued by Fujii et al. [13]. Zhang defines a notion of *graded category*, but it differs to ours, and is instead closer to a definition of a graded monad [55]. As we showed in Section 4, graded categories can be constructed both by monads and comonads graded by a pomonoid, and it can also capture graded structures that do not arise from either of them. Milius et al. [33] also studied monads graded by a pomonoid in the context of trace semantics where the grading represents a notion of depth corresponding to trace length. Exploring whether there is a generalization of our work to traces is an interesting future work.

Various works study comonads graded with a semiring structure as a semantic model of contextual computations captured by means of type systems [7,16,44]. In contrast, our graded comonads are graded by a pomonoid. The additive structure of the semiring in those works is needed to merge the gradings of different instances of the same variable. This is natural for the $\lambda$-calculus where the context represent multiple inputs, but there is only one conclusion (output). Here instead, we focus on an imperative language. So, we have only one input, the starting memory, and one output, the updated memory. Therefore, it is natural to have just the multiplicative structure of the semiring as a pomonoid. The categorical axiomatics of semiring-graded comonads are studied by Katsumata from the double-category theoretic perspective [25].

Apart from graded monads, several generalizations of monads has been proposed. Atkey introduces *parameterized monads* and corresponding *parameterized Freyd categories* [1], demonstrating that parameterized monads naturally model effectful computations with preconditions and postconditions. Tate defines *productors* with composability of effectful computations controlled by a relational 'effector' structure [53]. Orchard et al. define *category-graded monads*, generalizing graded and parameterised monads via lax functors and sketch a model of Union Bound Logic in this setting (but predicates and graded-predicate interaction are not modelled, as they are here) [41]. Interesting future work is to combine these general models of computational effects with Hoare logic.

### 7 Conclusion

We have presented a Graded Hoare Logic as a parameterisable framework for reasoning about programs and their side effects, and studied its categorical semantics. The key guiding idea is that grading can be seen as a refinement of effectful computations. This has brought us naturally to graded categories but to fully internalize this refinement idea we further introduced the new notion of graded Freyd categories. To show the generality of our framework we have shown how different examples are naturally captured by it.

We conclude with some reflections on possible future work.

*Future work* Carbonneaux et al. present a quantitative verification approach for amortized cost analysis via a Hoare logic augmented with multivariate quantities associated to program variables [8]. Judgments $\vdash \{\Gamma; Q\} S \{\Gamma'; Q'\}$ have pre- and post-conditions $\Gamma$ and $\Gamma'$ and potential functions $Q$ and $Q'$. Their approach can be mapped to GHL with a grading monoid representing how the potential functions change. However, the multivariate nature of the analysis requires a more fine-grained connection between the structure of the memory and the structure of grades, which have not been developed yet. We leave this for future work.

GHL allows us to capture the dependencies between assertions and grading that graded program logics usually use. However, some graded systems (e.g. [4]) use more explicit dependencies by allowing grade variables—which are also used for grading polymorphism. We plan to explore this direction in future work.

The setting of graded categories in this work subsumes both graded monads and graded comonads and allows flexibility in the model. However, most of our examples in Section 5.3 are related to graded monads. The literature contains various graded comonad models of data-flow properties: like liveness analysis [44], sensitivities [7], timing and scheduling [16], and information-flow control [40]. Future work is to investigate how these structures could be adopted to GHL for reasoning about programs.

*Acknowledgements* Katsumata and Sato carried out this research supported by ERATO HASUO Metamathematics for Systems Design Project (No. JPMJER1603), JST. Orchard is supported by EPSRC grant EP/T013516/1. Gaboardi is supported by the National Science Foundation under Grant No. 2040222.

## References

[1] Atkey, R.: Parameterised notions of computation. J. Funct. Program. **19**(3-4), 335–376 (2009). 10.1017/S095679680900728X

[2] Barthe, G., Gaboardi, M., Grégoire, B., Hsu, J., Strub, P.: Proving differential privacy via probabilistic couplings. In: 2016 31st Annual ACM/IEEE Symposium on Logic in Computer Science (LICS). pp. 1–10 (2016). 10.1145/2933575.2934554

[3] Barthe, G.: An introduction to relational program verification (2020), http://software.imdea.org/~gbarthe/__introrelver.pdf, working Draft

[4] Barthe, G., Gaboardi, M., Arias, E.J.G., Hsu, J., Roth, A., Strub, P.: Higher-order approximate relational refinement types for mechanism design and differential privacy. In: Rajamani, S.K., Walker, D. (eds.) Proceedings of the 42nd Annual ACM SIGPLAN-SIGACT Symposium on Principles of Programming Languages, POPL 2015, Mumbai, India, January 15-17, 2015. pp. 55–68. ACM (2015). 10.1145/2676726.2677000

[5] Barthe, G., Gaboardi, M., Grégoire, B., Hsu, J., Strub, P.: A Program Logic for Union Bounds. In: 43rd International Colloquium on Automata, Languages, and Programming, ICALP 2016, July 11-15, 2016, Rome, Italy. pp. 107:1–107:15 (2016). 10.4230/LIPIcs.ICALP.2016.107

[6] Barthe, G., Köpf, B., Olmedo, F., Zanella-Béguelin, S.: Probabilistic relational reasoning for differential privacy. ACM Trans. Progr. Lang. Syst. **35**(3), 9:1–9:49 (Nov 2013). 10.1145/2492061

[7] Brunel, A., Gaboardi, M., Mazza, D., Zdancewic, S.: A core quantitative coeffect calculus. In: Shao, Z. (ed.) Programming Languages and Systems - 23rd European Symposium on Programming, ESOP 2014, ETAPS 2014, Grenoble, France, April 5-13, 2014, Proceedings. Lecture Notes in Computer Science, vol. 8410, pp. 351–370. Springer (2014). 10.1007/978-3-642-54833-8_19

[8] Carbonneaux, Q., Hoffmann, J., Shao, Z.: Compositional certified resource bounds. In: Proceedings of the 36th ACM SIGPLAN Conference on Programming Language Design and Implementation, Portland, OR, USA, June 15-17, 2015. pp. 467–478 (2015). 10.1145/2737924.2737955

[9] Crole, R.L.: Categories for types. Cambridge University Press (1993)

[10] Day, B.: Construction of Biclosed Categories. Ph.D. thesis, School of Mathematics of the University of New South Wales (1970)

[11] Filinski, A.: Controlling Effects. Ph.D. thesis, Carnegie Mellon University (1996)

[12] Floyd, R.W.: Assigning meanings to programs. Proceedings of Symposium on Applied Mathematics **19**, 19–32 (1967). 10.1007/978-94-011-1793-7_4

[13] Fujii, S., Katsumata, S.y., Mellies, P.A.: Towards a formal theory of graded monads. In: International Conference on Foundations of Software Science and Computation Structures. pp. 513–530. Springer (2016). 10.1007/978-3-662-49630-5_30

[14] Gaboardi, M., Katsumata, S., Orchard, D., Sato, T.: Graded Hoare Logic and its Categorical Semantics. CoRR **abs/2007.11235** (2020), https://arxiv.org/abs/2007.11235

[15] Gaboardi, M., Katsumata, S., Orchard, D.A., Breuvart, F., Uustalu, T.: Combining effects and coeffects via grading. In: Garrigue, J., Keller, G., Sumii, E. (eds.) Proceedings of the 21st ACM SIGPLAN International Conference on Functional Programming, ICFP 2016, Nara, Japan, September 18-22, 2016. pp. 476–489. ACM (2016). 10.1145/2951913.2951939

[16] Ghica, D.R., Smith, A.I.: Bounded linear types in a resource semiring. In: Shao, Z. (ed.) Programming Languages and Systems - 23rd European Symposium on Programming, ESOP 2014, Held as Part of the European Joint Conferences on Theory and Practice of Software, ETAPS 2014, Grenoble, France, April 5-13, 2014, Proceedings. Lecture Notes in Computer Science, vol. 8410, pp. 331–350. Springer (2014). 10.1007/978-3-642-54833-8_18

[17] Gibbons, J.: Comprehending ringads - for phil wadler, on the occasion of his 60th birthday. In: Lindley, S., McBride, C., Trinder, P.W., Sannella, D. (eds.) A List of Successes That Can Change the World - Essays Dedicated to Philip Wadler on the Occasion of His 60th Birthday. Lecture Notes in Computer Science, vol. 9600, pp. 132–151. Springer (2016). 10.1007/978-3-319-30936-1_7

[18] Goncharov, S., Schröder, L.: A Relatively Complete Generic Hoare Logic for Order-Enriched Effects. In: 28th Annual ACM/IEEE Symposium on Logic in Computer Science, LICS 2013, New Orleans, LA, USA, June 25-28, 2013. pp. 273–282. IEEE Computer Society (2013). 10.1109/LICS.2013.33

[19] Goubault-Larrecq, J., Lasota, S., Nowak, D.: Logical relations for monadic types. Mathematical Structures in Computer Science **18**(6), 1169–1217 (2008). 10.1017/S0960129508007172

[20] Hasuo, I.: Generic weakest precondition semantics from monads enriched with order. Theoretical Computer Science **604**, 2 – 29 (2015). 10.1016/j.tcs.2015.03.047, coalgebraic Methods in Computer Science

[21] Ivašković, A., Mycroft, A., Orchard, D.: Data-Flow Analyses as Effects and Graded Monads. In: Ariola, Z.M. (ed.) 5th International Conference on Formal Structures for Computation and Deduction (FSCD 2020). Leibniz International Proceedings in Informatics (LIPIcs), vol. 167, pp. 15:1–15:23. Schloss Dagstuhl–Leibniz-Zentrum für Informatik, Dagstuhl, Germany (2020). 10.4230/LIPIcs.FSCD.2020.15

[22] Jacobs, B.: Categorical Logic and Type Theory. Elsevier (1999)

[23] Jacobs, B.: Dijkstra and Hoare monads in monadic computation. Theor. Comput. Sci. **604**, 30–45 (2015). 10.1016/j.tcs.2015.03.020

[24] Katsumata, S.: Parametric effect monads and semantics of effect systems. In: Jagannathan, S., Sewell, P. (eds.) The 41st Annual ACM SIGPLAN-SIGACT Symposium on Principles of Programming Languages, POPL ’14, San Diego, CA, USA, January 20-21, 2014. pp. 633–646. ACM (2014). 10.1145/2535838.2535846

[25] Katsumata, S.: A Double Category Theoretic Analysis of Graded Linear Exponential Comonads. In: Baier, C., Lago, U.D. (eds.) Foundations of Software Science and Computation Structures - 21st International Conference, FOSSACS 2018, ETAPS 2018, Thessaloniki, Greece, April 14-20, 2018, Proceedings. Lecture Notes in Computer Science, vol. 10803, pp. 110–127. Springer (2018). 10.1007/978-3-319-89366-2_6

[26] Katsumata, S., Sato, T., Uustalu, T.: Codensity lifting of monads and its dual. Logical Methods in Computer Science **14**(4) (2018). 10.23638/LMCS-14(4:6)2018

[27] Kura, S.: Graded Algebraic Theories. In: International Conference on Foundations of Software Science and Computation Structures. pp. 401–421. Springer (2020). 10.1007/978-3-030-45231-5_21

[28] Levy, P.B.: Locally graded categories. Slides available at http://www.cs.bham.ac.uk/~pbl/papers/locgrade.pdf (2019)

[29] Maillard, K., Ahman, D., Atkey, R., Martínez, G., Hritcu, C., Rivas, E., Tanter, É.: Dijkstra monads for all. Proc. ACM Program. Lang. **3**(ICFP), 104:1–104:29 (2019). 10.1145/3341708

[30] Maillard, K., Hritcu, C., Rivas, E., Muylder, A.V.: The next 700 relational program logics. Proc. ACM Program. Lang. **4**(POPL), 4:1–4:33 (2020). 10.1145/3371072

[31] Martin, U., Mathiesen, E.A., Oliva, P.: Hoare Logic in the Abstract. In: Ésik, Z. (ed.) Computer Science Logic. pp. 501–515. Springer Berlin Heidelberg, Berlin, Heidelberg (2006). 10.1007/11874683_33

[32] Melliès, P., Zeilberger, N.: Functors are Type Refinement Systems. In: Rajamani, S.K., Walker, D. (eds.) Proceedings of the 42nd Annual ACM SIGPLAN-SIGACT Symposium on Principles of Programming Languages, POPL 2015, Mumbai, India, January 15-17, 2015. pp. 3–16. ACM (2015). 10.1145/2676726.2676970

[33] Milius, S., Pattinson, D., Schröder, L.: Generic Trace Semantics and Graded Monads. In: Moss, L.S., Sobocinski, P. (eds.) 6th Conference on Algebra and Coalgebra in Computer Science (CALCO 2015). Leibniz International Proceedings in Informatics (LIPIcs), vol. 35, pp. 253–269. Schloss Dagstuhl–Leibniz-Zentrum fuer Informatik (2015). 10.4230/LIPIcs.CALCO.2015.253

[34] Moggi, E.: Notions of computation and monads. Inf. Comput. **93**(1), 55–92 (1991). 10.1016/0890-5401(91)90052-4

[35] Molnar, D., Piotrowski, M., Schultz, D., Wagner, D.A.: The program counter security model: Automatic detection and removal of control-flow side channel attacks. In: Won, D., Kim, S. (eds.) Information Security and Cryptology - ICISC 2005, 8th International Conference, Seoul, Korea, December 1-2, 2005, Revised Selected Papers. Lecture Notes in Computer Science, vol. 3935, pp. 156–168. Springer (2005). 10.1007/11734727_14

[36] Mycroft, A., Orchard, D.A., Petricek, T.: Effect Systems Revisited - Control-Flow Algebra and Semantics. In: Probst, C.W., Hankin, C., Hansen, R.R. (eds.) Semantics, Logics, and Calculi - Essays Dedicated to Hanne Riis Nielson and Flemming Nielson on the Occasion of Their 60th Birthdays. Lecture Notes in Computer Science, vol. 9560, pp. 1–32. Springer (2016). 10.1007/978-3-319-27810-0_1

[37] Nielson, H.R.: A Hoare-like proof system for analysing the computation time of programs. Science of Computer Programming **9**(2), 107–136 (1987). 10.1016/0167-6423(87)90029-3

[38] Nielson, H.R., Nielson, F.: Semantics with applications, vol. 104. Springer (1992)

[39] Olmedo, F.: Approximate Relational Reasoning for Probabilistic Programs. Ph.D. thesis, Technical University of Madrid (2014)

[40] Orchard, D., Liepelt, V., III, H.E.: Quantitative program reasoning with graded modal types. Proc. ACM Program. Lang. **3**(ICFP), 110:1–110:30 (2019). 10.1145/3341714

[41] Orchard, D., Wadler, P., III, H.E.: Unifying graded and parameterised monads. In: New, M.S., Lindley, S. (eds.) Proceedings Eighth Workshop on Mathematically Structured Functional Programming, MSFP@ETAPS 2020, Dublin, Ireland, 25th April 2020. EPTCS, vol. 317, pp. 18–38 2020). 10.4204/EPTCS.317.2

[42] Orchard, D.A., Petricek, T., Mycroft, A.: The semantic marriage of monads and effects. CoRR **abs/1401.5391** (2014), http://arxiv.org/abs/1401.5391

[43] Petricek, T., Orchard, D.A., Mycroft, A.: Coeffects: Unified static analysis of context-dependence. In: Fomin, F.V., Freivalds, R., Kwiatkowska, M.Z., Peleg, D. (eds.) Automata, Languages, and Programming - 40th International Colloquium, ICALP 2013, Riga, Latvia, July 8-12, 2013, Proceedings, Part II. Lecture Notes in Computer Science, vol. 7966, pp. 385–397. Springer (2013). 10.1007/978-3-642-39212-2_35

[44] Petricek, T., Orchard, D.A., Mycroft, A.: Coeffects: a calculus of context-dependent computation. In: Jeuring, J., Chakravarty, M.M.T. (eds.) Proceedings of the 19th ACM SIGPLAN international conference on Functional programming, Gothenburg, Sweden, September 1-3, 2014. pp. 123–135. ACM (2014). 10.1145/2628136.2628160

[45] Pitts, A.M.: Categorical logic. Tech. rep., University of Cambridge, Computer Laboratory (1995)

[46] Power, J.: Generic models for computational effects. Theoretical Computer Science **364**(2), 254–269 (2006). 10.1016/j.tcs.2006.08.006

[47] Power, J., Thielecke, H.: Environments, continuation semantics and indexed categories. In: Abadi, M., Ito, T. (eds.) Theoretical Aspects of Computer Software. pp. 391–414. Springer Berlin Heidelberg, Berlin, Heidelberg (1997)

[48] Sato, T.: Approximate Relational Hoare Logic for Continuous Random Samplings. In: Birkedal, L. (ed.) The Thirty-second Conference on the Mathematical Foundations of Programming Semantics, MFPS 2016, Carnegie Mellon University, Pittsburgh, PA, USA, May 23-26, 2016. Electronic Notes in Theoretical Computer Science, vol. 325, pp. 277–298. Elsevier (2016). 10.1016/j.entcs.2016.09.043

[49] Sato, T., Barthe, G., Gaboardi, M., Hsu, J., Katsumata, S.: Approximate Span Liftings: Compositional Semantics for Relaxations of Differential Privacy. In: 34th Annual ACM/IEEE Symposium on Logic in Computer Science, LICS 2019, Vancouver, BC, Canada, June 24-27, 2019. pp. 1–14 (2019). 10.1109/LICS.2019.8785668

[50] Smirnov, A.: Graded monads and rings of polynomials. J. Math. Sci. **151**(3), 3032–3051 (2008). 10.1007/s10958-008-9013-7

[51] Staton, S.: Freyd categories are Enriched Lawvere Theories. Electronic Notes in Theoretical Computer Science **303**, 197 – 206 (2014). 10.1016/j.entcs.2014.02.010, proceedings of the Workshop on Algebra, Coalgebra and Topology (WACT 2013)

[52] Staton, S.: Commutative semantics for probabilistic programming. In: Yang, H. (ed.) Programming Languages and Systems - 26th European Symposium on Programming, ESOP 2017, ETAPS 2017, Uppsala, Sweden, April 22-29, 2017, Proceedings. Lecture Notes in Computer Science, vol. 10201, pp. 855–879. Springer (2017). 10.1007/978-3-662-54434-1_32

[53] Tate, R.: The sequential semantics of producer effect systems. In: Giacobazzi, R., Cousot, R. (eds.) The 40th Annual ACM SIGPLAN-SIGACT Symposium on Principles of Programming Languages, POPL ’13, Rome, Italy - January 23 - 25, 2013. pp. 15–26. ACM (2013). 10.1145/2429069.2429074

[54] Wood, R.J.: V-indexed categories, chap. 2, pp. 126–140. No. 661 in Lecture Notes in Mathematics, Springer (1978). 10.1007/BFb0061362

[55] Zhang, J.J.: Twisted graded algebras and equivalences of graded categories. Proceedings of the London Mathematical Society **3**(2), 281–311 (1996). 10.1112/plms/s3-72.2.281

